# An experimental infection model for rapid reproduction of treponeme-associated hoof disease in captive elk (*Cervus canadensis*)

**DOI:** 10.1128/spectrum.03822-25

**Published:** 2026-04-09

**Authors:** Holly R. Drankhan, Kyle R. Taylor, Devendra H. Shah, Charlie H. Park, Alexis M. Grieser, Margaret A. Wild

**Affiliations:** 1 Department of Veterinary Microbiology and Pathology, College of Veterinary Medicine, Washington State University 70739, Pullman, Washington, USA; 2 Washington Animal Disease Diagnostic Laboratory, College of Veterinary Medicine, Washington State University 70739, Pullman, Washington, USA; 3 School of Veterinary Medicine, Texas Tech University, Amarillo, Texas, USA; Michigan State University, East Lansing, Michigan, USA

**Keywords:** treponeme-associated hoof disease, elk, *Treponema*, spirochetes, skin microbiome, skin scraping, 16S rRNA gene amplicon sequencing, infection model, anaerobic culture

## Abstract

Treponeme-associated hoof disease (TAHD) is an emerging, polybacterial infection that causes painful foot lesions and lameness in free-ranging elk (*Cervus canadensis*) across the northwestern USA. Although TAHD is associated with multiple *Treponema* species and other anaerobic bacteria, current understanding of disease etiology is limited and based primarily on cross-sectional analyses of naturally infected elk, in which lesions were examined at single time points using histopathology and 16S rRNA gene sequencing. Our objective was to develop a rapid and reliable experimental infection model to study TAHD pathogenesis in captive elk under controlled conditions. Inoculum consisting of macerated lesion tissue and mixed cultures of *Treponema* spp. and other anaerobic bacteria derived from TAHD lesions was applied onto abraded interdigital skin on the hind feet of five treatment elk. Inoculated feet were wrapped to expedite lesion development. Over the 7 week study period, all inoculated feet (10/10) developed interdigital erosions consistent with mild to moderate TAHD lesions observed in free-ranging elk. Lesions were observed on the majority of inoculated feet (9/10) after 28 days, coinciding with concurrent 16S rRNA gene amplicon sequencing detection of three putative pathogens of TAHD: *Treponema*, *Fusobacterium,* and *Mycoplasma*. In contrast, mock-inoculated control elk feet did not exhibit pathological or microbiological changes indicative of TAHD. This experimental infection model provides a valuable platform to investigate the complex interactions between the host, pathogens, and environmental factors that influence TAHD susceptibility, lesion progression, and disease transmission.

**IMPORTANCE:**

Our study details a new approach for consistent and rapid induction of treponeme-associated hoof disease (TAHD) lesions in captive elk. TAHD is an emerging polybacterial disease of conservation concern that causes chronic lameness and debilitation in free-ranging elk across the northwestern USA. We rapidly and reliably reproduced TAHD lesions following challenge with inoculum consisting of macerated lesion tissue and mixed cultures of *Treponema* spp. and other anaerobic bacteria. This experimental infection model provides a valuable platform for investigation of the complex interactions between the host, pathogens, and environmental factors influencing TAHD susceptibility, lesion progression, and disease transmission in elk.

## INTRODUCTION

Treponeme-associated hoof disease (TAHD) is an emergent disease affecting free-ranging elk (*Cervus canadensis*) within an expanding region of the northwestern USA that currently includes parts of Washington, Idaho, Oregon, and California (1). Affected elk develop lameness secondary to erosive or ulcerative interdigital dermatitis and pododermatitis, and overgrowth or sloughing of the hooves (2–4). TAHD lesions are classified into four macroscopic grades (I–IV), broadly reflecting progressive severity (3). Diagnostic confirmation is currently reliant upon histopathology, wherein TAHD-positive lesions are characterized by suppurative inflammation and identification of intralesional spirochetes using a Warthin-Starry or Steiner’s silver stain (1, 3, 5).

Molecular analyses have identified multiple *Treponema* phylogroups, including *T. phagedenis*, *T. medium/T. vincentii*, and *T. denticola/T. putidum* (now designated *T. pedis*), in TAHD lesions (1, 5–7). Similar *Treponema* phylogroups are also associated with bovine digital dermatitis (BDD) and contagious ovine digital dermatitis (CODD) lesions in domestic livestock (8–12). 16S rRNA and shotgun metagenomic sequencing studies have also identified novel *Treponema* genomospecies and unidentified *Spirochaetaceae* that may play a role in TAHD pathogenesis (1, 5, 13).

Microbiome profiling of TAHD, BDD, and CODD lesions indicates that these diseases likely have a complex polymicrobial etiology involving diverse *Treponema* spp., alongside other bacteria playing a role in precipitating or perpetuating disease, either as primary or opportunistic pathogens (5, 7, 14–17). These bacterial pathogens include *Mycoplasmataceae* (genus *Mycoplasma*) and anaerobes such as *Fusobacteriaceae* (genus *Fusobacterium*) and *Porphyromonadaceae* (genus *Porphyromonas*), which are detected more consistently and at a higher relative abundance in TAHD lesions (1, 5), as well as BDD lesions (15, 18–20), compared to unaffected feet. To date, there is no evidence that viruses or fungi play an etiologic role in TAHD (2, 3) or digital dermatitis in domestic ruminants (14). It has been proposed that poor nutrition, exposure to contaminants, and trace mineral deficiencies may influence TAHD susceptibility in wild elk populations (2, 3, 21, 22); however, these factors were not required for the development of TAHD in captive research elk (4).

Our current understanding of TAHD etiology is largely based on histopathologic examination coupled with 16S rRNA gene sequencing of naturally occurring TAHD lesions sampled postmortem. This approach has helped characterize the bacterial communities associated with TAHD lesions of different severity (5, 7), but it is unclear if these findings accurately reflect temporal microbiome shifts that occur over the course of lesion development in a single animal. The ability to perform longitudinal studies with captive animals under controlled conditions can help to overcome many constraints of disease investigations in naturally infected, free-ranging wildlife and is necessary to establish causation.

To date, only one published model has demonstrated that TAHD is transmissible to captive elk (4). Although successful in meeting the study objective of determining environmental transmission and establishing TAHD as an infectious and transmissible disease, the model required a prolonged challenge period and a large quantity of tissue sourced from TAHD-positive feet of multiple elk to prepare inocula consisting of macerated tissue and soil (4). Incorporation of soil into the inoculum also introduced inherent variability. In a separate study, erosive foot lesions were more rapidly induced in domestic sheep following a single exposure to TAHD lesion material (23). While informative, this model is constrained by its reliance on a non-target species and production of lesions that differed in both anatomical location and gross appearance from those observed in elk. Therefore, a need remains for a refined experimental TAHD infection model in elk that is less time- and resource-intensive to facilitate replication. Models developed for rapid reproduction of digital dermatitis in livestock incorporate manual abrasion of the skin to facilitate pathogen entry, use of inoculum enriched for putative pathogens, and wrapping of inoculated feet to create a moist, low-oxygen microenvironment conducive to anaerobic bacterial growth, highlighting the complexity of successful experimental transmission of these infections (24–27).

The objective of this study was to develop an experimental model for the rapid and consistent induction of TAHD lesions in captive elk. We hypothesized that captive elk exposed to a combination of macerated TAHD lesion tissue and cultures enriched for cultivable *Treponema* spp. and other anaerobic bacteria would rapidly develop gross and histologic lesions consistent with those observed in naturally infected free-ranging elk.

## MATERIALS AND METHODS

### Experimental design and captive elk management

**Fig 1:**
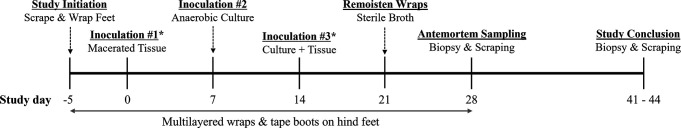
Timeline of 7 week study period to develop an experimental transmission model for treponeme-associated hoof disease in captive elk. Asterisks indicate time points when foot wraps were entirely removed for gross examination and collection of a skin scraping prior to inoculum application.

Seven 9-month-old female Rocky Mountain elk (*Cervus canadensis nelsoni*) were obtained from Starkey Experimental Forest and Range in northeastern Oregon and transported to the Washington State University Elk Research Facility (Pullman, WA, USA). Elk were provided a maintenance diet consisting of grass hay and pelleted feed (Mazuri Exotic Animal Nutrition, St. Louis, MO, USA), with *ad libitum* access to water and mineral salt blocks. At enrollment in this study, elk were 2 or 3 years old and individually housed in partially covered outdoor pens (~31 m^2^) with concrete floors covered in wood shavings. Elk were randomly assigned to either a treatment group (*n* = 5) or a control group (*n* = 2). Following a 4 week acclimation period, experimental procedures for both groups were conducted concurrently during October and November 2024. During the study period, both hind feet (RH, right hind foot; LH, left hind foot) of each elk were repeatedly examined, abraded, inoculated, and wrapped (Fig. 1). Food was withheld for approximately 24–36 hours prior to each procedure for which elk were immobilized with an intramuscular injection of butorphanol, azaperone, and medetomidine (Wedgewood Pharmacy, Swedesboro, NJ, USA).

### Preparation of inoculum

To optimize the likelihood of rapid reproduction of TAHD lesions, we applied two forms of inoculum at three time points. Treatment inoculum was prepared using a total of seven feet from four free-ranging elk harvested or culled by wildlife management agencies in regions of western Washington with previous laboratory-confirmed TAHD cases (Table S1). Feet were stored at −80°C within 24 hours of death, then thawed for inoculum preparation as previously described (4). Surface debris was removed with deionized (DI) water, then feet were assigned a TAHD lesion grade using a published grading scheme (3). Skin biopsies (5 mm and 8 mm) were aseptically collected from the interdigital space and/or coronary band of each foot selected for inoculum preparation using disposable biopsy punches (Integra Miltex, Princeton, NJ, USA). The 5 mm biopsies were preserved in Allprotect Tissue Reagent (QIAGEN, Germantown, MD, USA) at −80°C for DNA extraction. The 8 mm punch biopsies were fixed in 10% neutral-buffered formalin (NBF) for histologic assessment. Two forms of treatment inoculum were prepared from the remaining interdigital tissue: (i) macerated lesion tissue and (ii) anaerobic enrichment culture.

#### Macerated lesion tissue-based inoculum

The first treatment inoculum, prepared and applied on day 0 (Fig. 1), consisted of 16.4 g of total interdigital tissue aseptically collected from three feet with TAHD lesion grades III–IV and one grade 0 foot with mild gross changes (Table S1). The tissues were transported anaerobically (AnaeroPack, Mitsubishi Gas Chemical, Tokyo, Japan) and then processed in an anaerobic chamber (BACTRONEZ, Sheldon Manufacturing, Cornelius, OR, USA) supplied with anaerobic mixed gas (90% N, 5% H, 5% CO_2_). Tissues were minced into 2–3 mm^3^ pieces, then pooled and divided into 10 aliquots, each consisting of approximately 2 mL macerated tissue in 5 mL of MTGE broth (Anaerobe Systems, Morgan Hill, CA, USA).

#### Anaerobic enrichment culture-based inoculum

The second treatment inoculum, applied on day 7 (Fig. 1), consisted of mixed anaerobic enrichment cultures derived from grade II and grade III TAHD lesions (Tables S1 and S2). Techniques used to prepare the enrichment cultures were adapted from published methods for isolating *Treponema* sp. from TAHD lesions in elk (6), CODD lesions in sheep (11), and BDD lesions in cattle (8, 28–30). Rather than establishing pure cultures, we aimed to enrich for multiple fastidious treponemes and other obligate and facultative anaerobic bacteria implicated as putative TAHD pathogens while suppressing contaminant and commensal bacteria. Cultures were initiated in oral treponeme enrichment broth (OTEB, Anaerobe Systems, Morgan Hill, CA, USA) supplemented with 10% (vol/vol) rabbit serum (RS) or heat-inactivated fetal bovine serum (FBS; Gibco, Grand Island, NY, USA), and antibiotics (5 μg/mL enrofloxacin HCl and 10 μg/mL rifampicin; Toku-E, Bellingham, WA, USA). Multiple 10 mL vials of culture media were each inoculated with minced tissue from two 5 mm punch biopsies aseptically collected from TAHD lesions and stored in vials of Anaerobic Transport Medium (Anaerobe Systems, Morgan Hill, CA, USA) at room temperature for ≤26 hours. Cultures were incubated anaerobically at 37°C with periodic assessment of wet mounts prepared from 7 µL of undiluted culture media via darkfield microscopy.

**Fig 2:**
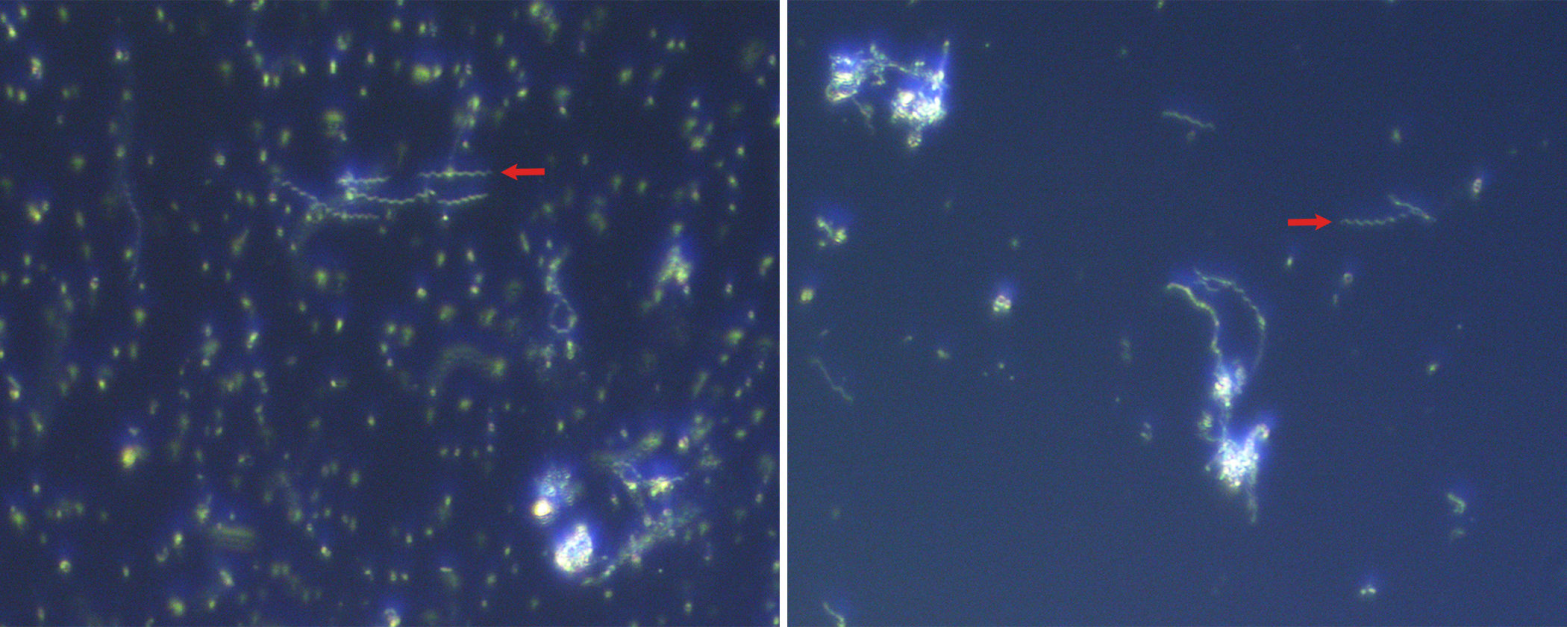
Wet mount preparations of mixed anaerobic cultures used to prepare the treatment inoculum were examined by darkfield microscopy to identify spirochete bacteria (red arrows). Images were captured using a digital microscope-mounted camera with an attached 0.5× reduction lens and a 40× objective lens. Both images were similarly cropped and enlarged to improve detail.

On day 7 of the study, five enrichment cultures (incubated for 5.5 or 8.5 days) that exhibited dense bacterial populations with diverse morphologies, including slender, spiral-shaped bacteria resembling spirochetes (Fig. 2) with corkscrew-like motility (estimated average 8–24 per 400× field), were selected for inoculum preparation (Table S2). Approximately 5 mL from each selected culture was combined with 30 mL sterile OTEB and divided into 10 5 mL aliquots for inoculation.

#### Combined culture and macerated tissue-based inocula

A combination of anaerobic cultures and macerated elk hoof tissue was used for the day 14 treatment inoculum (Fig. 1). We prepared subcultures of the grade II TAHD lesion cultures used in the day 7 inoculum by inoculating 250 µL of the 4-day-old enrichment culture into fresh OTEB supplemented with serum and antibiotics. For the day 14 inoculum, 5 mL aliquots from two of the subcultures (approximately 11.5 days post-incubation) and one primary culture of the grade III lesion (approximately 12.5 days post-incubation; Table S2) were combined with an additional 20 mL sterile OTEB, then divided into ten, 3 mL inoculum doses. To supplement the culture-based inoculum, we also prepared a new batch of macerated tissue inoculum (Table S1), which was divided into aliquots containing 1 mL of pooled minced tissue and 3 mL MTGE broth.

#### Control inoculum

Control macerated tissue was prepared aerobically from elk feet without apparent gross lesions. Punch biopsies were collected for histologic assessment of each foot, as described for the treatment inoculum. Aliquots of macerated control tissue and MTGE broth were autoclaved (121°C for 45 minutes) and stored at −80°C. On days 0 and 14, control tissue was thawed and applied to the hind feet of control elk (2 mL or 1 mL macerated tissue per foot, respectively). On days 7 and 14, sterile OTEB was applied onto the hind feet of the control elk in place of anaerobic cultures.

We characterized the microbial composition of the inocula using two subsamples from each batch of macerated tissue that were preserved in Allprotect and bacterial cell pellets that were obtained by centrifuging aliquots of each culture (1 mL) and final culture-based inocula (2 mL) and resuspended in 800 µL 1× DNA/RNA Shield (Zymo Research, Irvine, CA, USA). Samples were frozen at −80°C for subsequent DNA extraction and sequencing.

### Application of foot wraps and inoculum

**Fig 3:**
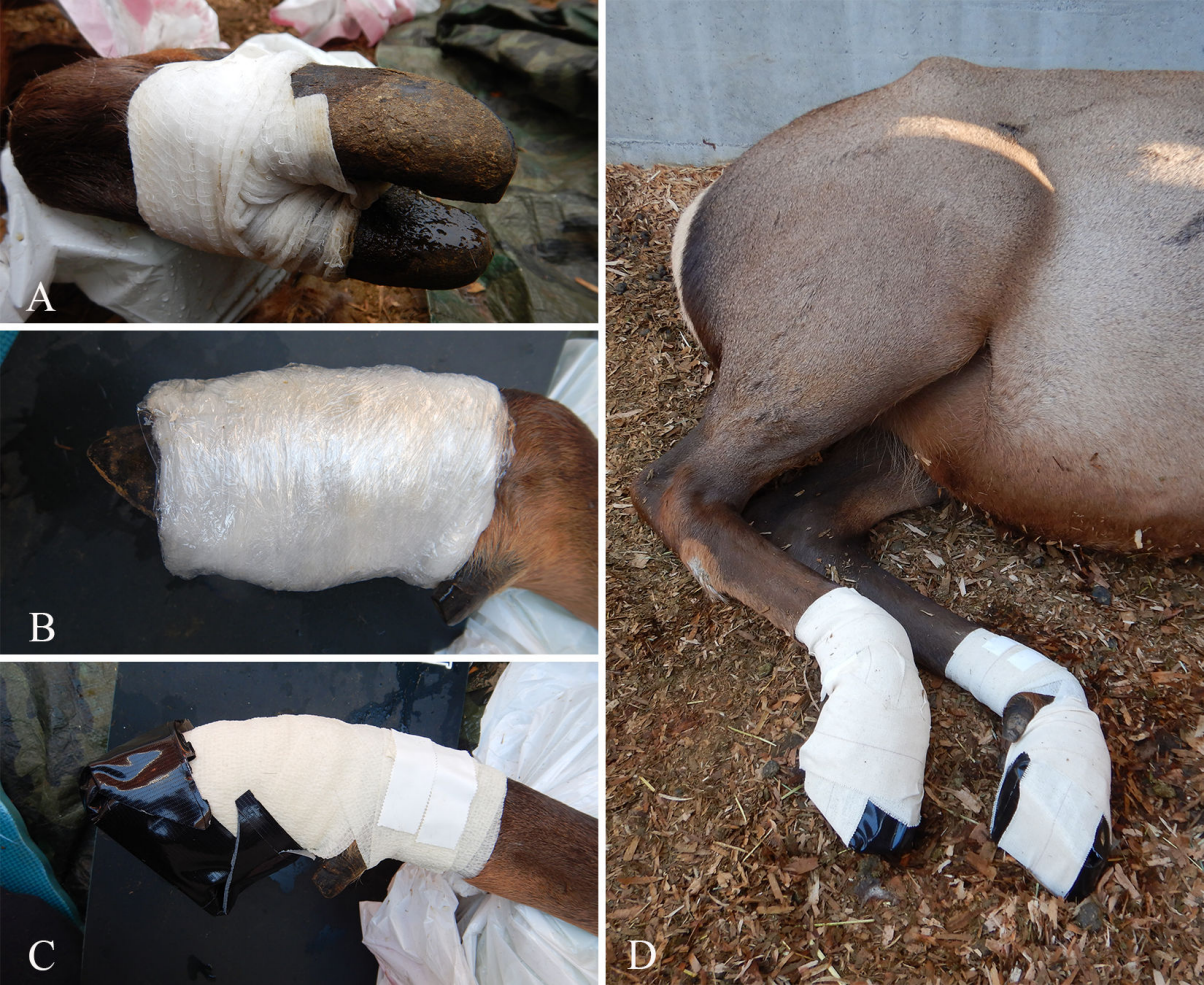
Pictorial summary of foot wraps applied to captive elk for experimental transmission of treponeme-associated hoof disease. (**A**) A woven gauze pad containing inoculum and broth was placed on the interdigital skin and covered by roll gauze. (**B**) Absorbent, water-soaked cotton padding, was placed around the distal limb and covered by plastic stretch wrap to hold in moisture. (**C and D**) Outer layers of elastic bandage wrap, elastic tape, and Gorilla tape provided a protective barrier and secured wraps to the hind feet.

Five days before inoculation (designated day –5; Fig. 1), all four feet from each elk were rinsed with DI water and visually inspected to confirm the absence of gross abnormalities. On both hind feet, an approximately 2 cm^2^ area of interdigital skin was abraded using a sterile #21 scalpel blade until light bleeding occurred. Following abrasion, moist multilayered wraps were applied to the hind feet (Fig. 3), incorporating elements from previously successful BDD and TAHD infection models (4, 23–27). The abraded interdigital skin was covered with a 3 × 3 inch sterile woven gauze pad secured with a roll of sterile, 2-inch-wide conforming gauze bandage (Medline Industries, Northfield, IL, USA) wrapped between and around the toes and fastened with waterproof tape. Next, water-soaked Gamgee Highly Absorbent Padding (3M, Maplewood, MN, USA) was applied circumferentially below the dewclaws and covered with six layers of plastic stretch wrap to hold in moisture. These inner wrap layers were covered with self-adherent elastic bandage wrap (Cohesiant Wrap, AmerisourceBergen, Conshohocken, PA, USA) and anchored to the metatarsal skin with elastic tape (Elastikon, Actimove, Essity, Stockholm, Sweden). A water-saturated absorbent fiber pad (Therapeutic Pads, DAVIS Plastics LLC, Brandon, WI, USA) was applied to the sole, then the hoof was covered with an outer tape boot composed of four layers of overlapping Gorilla tape strips (Gorilla Glue, Cincinnati, OH, USA) and secured to the underlying wrap layers with additional elastic tape.

Aliquots of treatment inocula (macerated tissue and/or culture), transported and maintained anaerobically in an Anaeropack system, were applied to the hind feet of treatment elk on days 0, 7, and 14 (Fig. 1). On the same days, control animals were mock-inoculated with similar volumes of sterile OTEB and/or control macerated tissue. On days 0 and 14, existing wraps were entirely removed from the hind feet of both treatment and control animals, and the interdigital skin was re-abraded as detailed in the subsequent section. Then, aliquots of treatment or control macerated tissue inoculum were applied onto a new sterile woven gauze pad that was placed over the abraded interdigital skin and secured with rolled gauze. On day 7, the tape boots and outer wrap layers were removed, but intact inner gauze layers containing previously applied macerated tissue were left in place to avoid disrupting lesion development. We infused 5 mL of mixed anaerobic cultures (treatment group) or sterile broth (control group) behind the wraps onto the interdigital skin using a syringe with an attached 1.3-inch sterile plastic teat cannula (Jorgensen Laboratories, Loveland, CO, USA). A similar method was also used to administer aliquots of mixed anaerobic cultures (treatment group) or sterile broth (control group) following application of macerated tissue inoculum on day 14.

No inoculum was applied on day 21. Instead, elk in both groups had all components of the existing wraps removed and replaced, except the interdigital gauze pads containing the day 14 inoculum. We infused 5 mL of sterile broth (2.5 mL each MTGE and OTEB) behind this layer to keep the interdigital skin moist.

Following application of inoculum or sterile broth on days 0, 7, 14, and 21, additional OTEB or MTGE broth was used to moisten the inner gauze layers, and the hind feet were rewrapped and covered with new tape boots as previously described for day –5 and illustrated in Fig. 3.

### Antemortem monitoring and sampling

The front and hind feet of all elk were examined for gross lesions on days 0, 14, and 28. At these time points, the hind feet were unwrapped, and the interdigital skin was clipped, swabbed with a cotton-tipped applicator, and rinsed with purified water (DI or distilled). A veterinarian not blinded to treatment group assigned an apparent gross lesion grade to hind feet based on criteria from the published TAHD grading scheme (3). Feet without any apparent lesions on the interdigital skin were assigned grade 0. Discrete cutaneous erosions confined to interdigital skin were designated grade I lesions. Lesions were classified as grade II if there was an active interdigital erosion extending to the coronary band and undermining or disrupting the adjacent hoof capsule and/or heel bulb. If there were multiple separate lesions on a single foot, grading was based on the most severe lesion observed. Characteristics of grade III and IV TAHD lesions in free-ranging elk (i.e., sole ulcers, broken or sloughed hoof capsules) were not observed during the course of this study.

On days 0 and 14, skin scrapings were collected from the hind feet of all elk using a sterile #21 scalpel blade. On day 0, the skin was scraped in the same location as on day −5. On day 14, skin scrapings were collected from the margins of any apparent interdigital cutaneous erosions or at the same location as previous scrapings if no gross lesions were observed. The scrapings were stored in sterile microcentrifuge tubes at −80°C for DNA extraction.

Paired antemortem biopsies and scrapings of interdigital skin were aseptically collected from both hind feet of all elk on day 28, as described by Robinson et al. (4). We sampled the margins of any grossly apparent erosions unless precluded by proximity to the hoof capsule. Biopsies (5 mm) were fixed whole in 10% NBF for histologic examination to confirm whether observed lesions were microscopically consistent with TAHD, and paired scrapings were frozen at −80°C for DNA extraction. Following sample collection, the hind feet were wrapped with fewer layers of sterile gauze, self-adherent elastic bandage wrap, and Gorilla tape to temporarily cover the biopsy sites, which were allowed to heal by second intention. A single dose of meloxicam (about 1 mg/kg; Zydus Pharmaceuticals USA Inc, Pennington, NJ, USA) was administered orally to all elk on day 28 to mitigate pain associated with biopsy collection.

One treatment elk (22-04) developed a consistent limp beginning on day 36 of the study, characterized by a shortened stride and reduced weight bearing on the RH limb when in motion. For the last 6 days of the study, this elk received meloxicam (about 1 mg/kg) in an apple treat once daily to mitigate pain and discomfort. No other treatment or control elk exhibited significant and consistent lameness warranting analgesic treatment per the approved protocol.

### Study conclusion

All treatment elk (5/5) and one control elk (1/2) were euthanized between study days 42 and 44 via intravenous injection of Euthasol (pentobarbital sodium and phenytoin sodium, Virbac, Westlake, TX, USA). Complete necropsies were subsequently performed at the Washington Animal Disease Diagnostic Laboratory (WADDL). All four feet were examined and assigned a gross lesion grade, as previously described. We aseptically collected 5 mm punch biopsies and paired scrapings from the interdigital skin on both front and hind feet of all elk and stored these samples dry (scrapings) or in Allprotect (biopsies) at −80°C for DNA extraction. The remaining interdigital skin was excised from each foot and fixed in 10% NBF for histologic examination. Representative samples of the viscera were also collected in 10% NBF to examine for microscopic lesions in other tissues.

For one treatment elk (22-04), a bandsaw was used to sagittally section the distal phalanx, sole, and hoof capsule of the RH lateral digit due to the detection of sole separation along the white line. Samples of the epidermis and corium of the hoof and sole at the tip of the affected digit were collected in 10% NBF for histopathology. Additional fresh samples of these tissues were also collected for DNA extraction after the foot was frozen at −20°C for approximately 5 months and thawed in a 37°C water bath.

One control elk (22-01) was not euthanized for humane reasons. Instead, this animal was immobilized on day 41 for final foot examination and sampling. A 5 mm punch biopsy and paired scraping were collected from the interdigital skin of all four feet. Each punch biopsy was bisected aseptically; one half was placed in 10% NBF for histology, and the other was stored at −80°C in Allprotect for DNA extraction.

### Histopathology

A board-certified veterinary pathologist performed histologic examination of bisected, formalin-fixed punch biopsies collected from all feet used to prepare inocula and from the feet of captive elk on day 28. Pathologists also examined a bisection of each endpoint foot biopsy from the control elk that was not euthanized (22-01) and two to five representative sections of the interdigital skin collected postmortem from the euthanized treatment and control elk. The pathologists were blinded to 16S amplicon sequencing results but not study group and trimmed tissues from each foot with knowledge of its gross appearance to obtain appropriate representative sections for comprehensive histologic assessment. Additionally, pathologists examined representative sections of the viscera collected from all euthanized elk and sections of abnormal hoof laminae from elk 22-04.

Tissue sections were routinely processed and embedded in paraffin, then sectioned and stained with hematoxylin and eosin (H&E) following WADDL’s standard operating procedures. Warthin-Starry silver-stained sections of interdigital skin from the hind feet of captive elk and feet used to prepare the treatment inocula were also examined for intralesional argyrophilic (i.e., silver stain-positive) spirochetes. Tissue sections stained with Brown-Hopps Gram stain were evaluated as needed to further differentiate the bacteria within cutaneous lesions (1). Per established criteria (3, 5), feet were considered TAHD-positive if suppurative inflammation and intralesional argyrophilic spirochetes were identified in examined histologic sections.

### Anaerobic culture of an experimentally induced lesion

Using culture methods similar to those described for inoculum preparation, we attempted to recultivate spirochetes and other putative bacterial pathogens from biopsies of the largest and most severe experimentally induced TAHD lesion observed at the conclusion of the study: a grade II lesion on the LH foot of elk 23-10 (Table 1). Each culture vial was inoculated with a single minced 5 mm punch biopsy. At 2 and 6 days post-incubation, wet mounts were examined via darkfield microscopy, and cell pellets prepared from 2 mL aliquots were stored for DNA extraction as described previously.

**TABLE 1:** Gross lesion grades, histologic confirmation of TAHD, and detection of intralesional treponemes via 16S rRNA gene amplicon sequencing at each post-inoculation examination*^f^*^*,^g^,^h^*^

			Day 14		Day 28			Days 41–44		
	Elk ID	Foot	Grade	16S^Sc^	Grade	16S^Sc^	Histo	Grade	16S^Sc/Bx^	Histo
	22-03	RH	**II**	−	**I**	**+**	**+**	0^*a*^	**+/+**	**+**
Treatment		LH	**I**	**+**	**I**	**+**	**+**	0^*a*^	**+/+**	**+**
	22-04	RH	0	−	0	−	−	**I** ^*b*^	−**/+**	**+**
		LH	0	−	**II**	**+**	−	0	**+/**−	−
	23-10	RH	**II** ^*c*^	**+**	**I** ^*c*^	**+**	**+**	0^*a*^	**+/+**	**+**
		LH	**I**	−	**II** ^*c*^	**+**	−^*d*^	**II** ^*c*^	**+/+**	**+**
	23-11	RH	**I**	NA	**I**	**+**	**+**	0^*a*^	**+/+**	**+**
		LH	**I**	−	**I**	**+**	**+**	0^*a*^	**+/+**	**+**
	23-16	RH	**II**	**+**	**II**	**+**	**+**	**I**	−/**+**	−
		LH	**I**	**+**	**I**	**+**	−	**I**	**+/+**	**+**
Control	22-01	RH	0	NA	0	−	−	0^*a*^	−/−	−
		LH	0	NA	0	−	−	0^*a*^	−/−	−
	23-14	RH	0	−	0	−	−	0^*a*^	−/−	−^*e*^
		LH	0	−	0	−	−	0	−/−	−

^*a*^ Gross lesions on interdigital skin restricted to healing antemortem biopsy site, so assigned gross lesion grade 0.

^*b*^ In addition to a grade I cutaneous lesion, this foot also had a necrotizing lesion at the distal aspect of the lateral digit (see Fig. 7).

^*c*^ Two discrete interdigital erosions were detected on the same foot, and grade was assigned based on the most severe lesion.

^*d*^ Cutaneous ulcer with suppurative inflammation detected histologically, but no apparent intralesional spirochetes.

^*e*^ Minimal intraepidermal suppurative inflammation detected histologically at previous biopsy site, but no associated erosion or intralesional spirochetes.

^*f*^ Grade: Gross lesion grade (0–II) assigned to each hind foot based on published TAHD grading scheme (3). Grade 0 = no gross lesions observed on the interdigital skin. Grade I = discrete cutaneous erosion. Grade II = cutaneous erosion extending to coronary band and undermining hoof capsule or heel bulb. 16S: Detection of genus *Treponema* in foot scrapings (Sc) and biopsies (Bx) via 16S rRNA gene amplicon sequencing, based on taxonomic assignments using the Zymo Research reference database. Results reported as detected (+) or not detected (−) for each sample. NA = 16S sequencing results not available. Histo: Histologic diagnosis of TAHD based on identification of suppurative inflammation and intralesional argyrophilic spirochetes in sections of interdigital skin. Results reported as TAHD-positive (+) or TAHD-negative (−) for each foot.

^*g*^ Results are reported separately for each inoculated hind foot from all captive elk (RH, right hind foot; LH, left hind foot).

^*h*^ Bolded text used to emphasize feet with gross and/or histologic TAHD lesions and *Treponema *detection.

### DNA extraction and 16S rRNA gene amplicon sequencing

Genomic DNA was extracted from biopsies of feet used to prepare the treatment inoculum, subsamples of pooled macerated tissue inocula, and skin biopsies and scrapings from study elk using the ZymoBIOMICS DNA Miniprep Kit (Zymo Research, Irvine, CA, USA), following published protocols (1, 4). Bacterial pellets from enrichment cultures preserved in DNA/RNA Shield were submitted to Zymo Research for mechanical homogenization and DNA purification using the ZymoBIOMICS MagBead DNA/RNA Kit (Zymo Research). The V3–V4 region of the 16S rRNA gene was PCR-amplified and sequenced by the Zymo Research Microbiome Sequencing Service, with downstream processing and taxonomic assignments performed as previously described (1, 5).

Microbial community composition and relative abundance (RA) of bacterial taxa were assessed by comparing read counts within the kingdom Bacteria. Analyses focused on bacterial taxa commonly implicated in TAHD: *Spirochaetaceae* (genus *Treponema* and unidentified *Spirochaetaceae*), *Fusobacteriaceae* (genus *Fusobacterium*), and *Mycoplasmataceae* (genus *Mycoplasma*). Reads within family *Spirochaetaceae* were further examined to resolve *Treponema* phylogroups. Amplicon sequence variants (ASVs) classified as *T. medium* or *T. medium-vincentii* and *T. denticola* or *T. denticola-putidum* were grouped to align with established BDD *Treponema* phylogroups (8). Unidentified *Spirochaetaceae* sequences were queried by BLAST against the National Center for Biotechnology Information database for additional taxonomic resolution. For elk with multiple postmortem samples per hind foot, analyses prioritized paired interdigital biopsies and scrapings from gross or histologic lesions, or from sites where *Treponema* spp. were detected.

Alpha diversity was assessed for captive elk hind foot samples using the Shannon diversity index (SDI) in Qiime v.1.9.1 (31), with rarefaction to 20,000 sequences per sample. Each hind foot was treated as an individual sampling unit. SDI values were compared between treatment and control elk at days 0, 28, and 41–44 using Mann-Whitney *U* tests. We also assessed differences in the SDI of treatment elk foot scrapings between time points using a Friedman test and *post hoc* pairwise comparisons using Wilcoxon signed-rank test with Bonferroni correction, which are non-parametric tests suitable for repeated measures. Assessment for differences between time points was not conducted for control elk scrapings due to the small sample size (*n* = 4). Statistical analyses were performed using R version 4.5.1 and R Studio version 2025.09.1+401.

### Photomicrographs and gross images

All figures with photomicrographs and gross images were assembled in Photoshop, with cropping as needed to emphasize areas of interest. All brightfield photomicrographs were white balanced.

## RESULTS

### Inoculum assessment

#### Histopathology and microbial composition of feet sampled for treatment inocula

Histologic assessment of grade II–IV lesions used to prepare the day 0 treatment inoculum and anaerobic enrichment cultures identified suppurative inflammation with intralesional argyrophilic spirochetes, confirming TAHD-positive status (Table S1). 16S rRNA amplicon sequencing detected multiple *Treponema* spp. and other unidentified *Spirochaetaceae* in biopsies from these feet, with a high proportion of *T. pedis* (Table S1). TAHD was also confirmed histologically for one of the two feet used for the day 14 macerated tissue inoculum, and *Spirochaetaceae* sp66598 was the only *Spirochaetaceae* detected in a biopsy from this foot via sequencing (Table S1). *Fusobacterium* and *Mycoplasma* were also detected by sequencing in biopsies from most of the TAHD-positive feet used to prepare the treatment inoculum (Table S1).

#### Anaerobic enrichment cultures

Cell pellets of enrichment cultures prepared from the grade II TAHD lesion and used in treatment inocula on days 7 and 14 were dominated by anaerobes *Fusobacteriaceae* and *Clostridiales* Family XII (82.7%–96.7% combined RA; Table S2). The two cultures of the grade III TAHD lesion varied in bacterial composition, likely reflecting differences in culture duration and serum supplementation. *Erysipelotrichaceae* and *Clostridiales* Family XI predominated (RA = 63.7% and 29.3%, respectively) in the culture supplemented with RS and incubated for 5.5 days, whereas *Porphyromonadaceae* (RA = 44.5%) and *Fusobacteriaceae* (RA = 33.4%) predominated in the culture supplemented with FBS and incubated for 12.5 days (Table S2).

*Spirochaetaceae* was detected in all enrichment cultures used for inoculum preparation (RA = 0.5%–6.5%; Table S2). *T. phagedenis* was the predominant treponeme in enrichment cultures of the grade II TAHD lesion, regardless of serum supplementation (>97% of *Spirochaetaceae* reads; Table S2). *T. medium* and *T. medium-vincentii* were detected only in cultures of the grade III TAHD lesion, comprising >98% of *Spirochaetaceae* reads in RS-supplemented cultures incubated for 5.5 days (Table S2). *T. pedis* and *Spirochaetaceae* sp66653 were detected inconsistently and at low RA in the enrichment cultures (Table S2), despite representing a high proportion of the *Spirochaetaceae* reads in biopsies of the cultured lesions (Table S1). As expected, *Mycoplasmataceae* was not detected in any enrichment cultures.

#### Treatment inoculum

**Fig 4:**
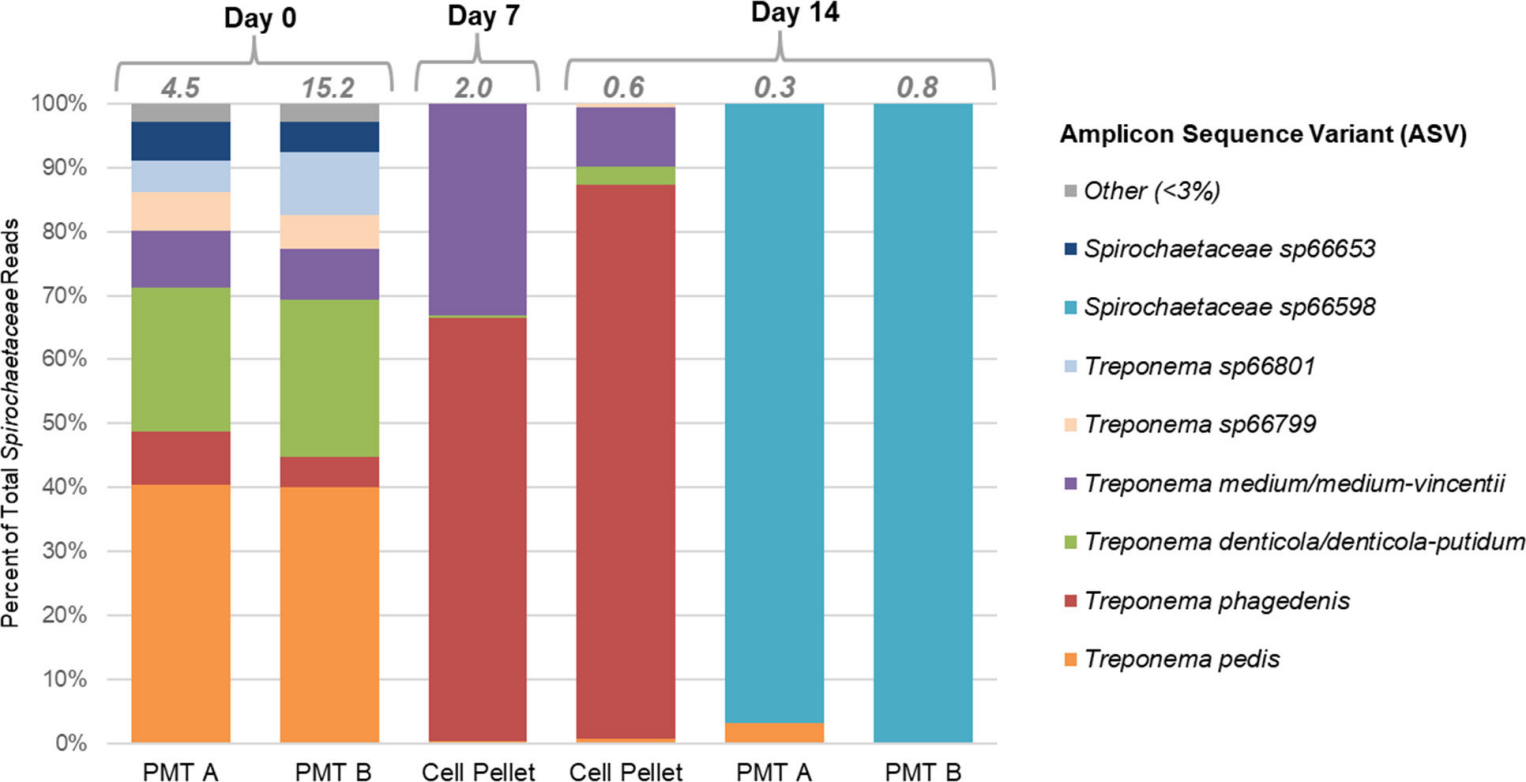
Relative proportion of ASVs within family *Spirochaetaceae* detected in each sample of treatment inoculum, including PMT (subsamples A and B) applied on days 0 and 14 and culture-based inocula (cell pellets) applied on days 7 and 14. “Other” includes ASVs that comprised <3% of total *Spirochaetaceae* reads in the samples of day 0 PMT and were not detected in the other inoculum samples. The total relative abundance of family *Spirochaetaceae* in each sample is indicated with italicized gray numbers at the top of each bar.

Both subsamples of the pooled macerated tissue inoculum (PMT A and PMT B) applied on day 0 contained *Spirochaetaceae* (RA = 4.5% or 15.2%; Table S3), including unidentified *Spirochaetaceae*, *T. pedis, T. phagedenis, T. medium/medium-vincentii, T. denticola/denticola-putidum,* and ASVs with high sequence similarity to *T. medium* (*Treponema* sp66799 and *Spirochaetaceae* sp66653; Fig. 4; Table S4). These align with *Treponema* spp. previously reported to be associated with TAHD (4–7). In contrast, macerated tissue inoculum prepared on day 14 contained markedly lower RA of *Spirochaetaceae* (≤0.8%), predominated by *Spirochaetaceae* sp66598 (Fig. 4; Table S3).

*Spirochaetaceae* were detected in the enrichment culture-based inocula applied on days 7 and 14 (RA = 2.0% and 0.6%, respectively), predominantly *T. phagedenis* and *T. medium/medium-vincentii* (Fig. 4; Table S3). Both culture-based inocula also contained high RA of *Fusobacteriaceae* (69.9% and 52.2% on days 7 and 14, respectively; Table S3). *Mycoplasma* (*Mycoplasmataceae*) was a minor component of the day 0 inoculum (RA ≤0.3%) and was not detected in the day 7 or day 14 culture-based inocula or subsamples of the day 14 macerated tissue inoculum (Table S3).

#### Control inoculum

The feet used for control inoculum were grossly and histologically normal (TAHD-negative). 16S sequencing detected no *Mycoplasma* or *Fusobacterium* spp. and low RA *Spirochaetaceae* (0.2%) in subsamples of the control inoculum. Aliquots of control inoculum were autoclaved before use.

### Integrity of foot wraps

#### Pre-inoculation period

Except for the right hind feet of one treatment elk (22-04) and one control elk (22-01), all feet remained wrapped during the 5 day pre-inoculation period. After 5 days, the feet that remained encased by water-soaked wraps had moist and soft interdigital skin.

#### Post-inoculation period

Wraps remained intact on both hind feet of most treatment elk (4/5) from day 0 through day 21, except during planned wrap changes. One treatment elk (22-04) repeatedly lost wraps on one or both hind feet 4–5 days after each inoculation, and the uncovered feet were rewrapped at the next planned immobilization. Both hind feet of all treatment elk remained wrapped from day 21 through day 28.

Each control elk lost one wrap shortly after application of sterile broth on day 7. Replacement wraps were applied within 20 hours (22-01) or on day 14 (23-14). Wraps remained intact for both control elk from day 14 through day 28, and, importantly, one hind foot from each control elk remained continuously wrapped from day −5 through day 28, except during planned wrap changes. By day 34, all elk lost one or both of the wraps applied post-biopsy.

### Gross and histologic assessment of captive elk feet

**Fig 5:**
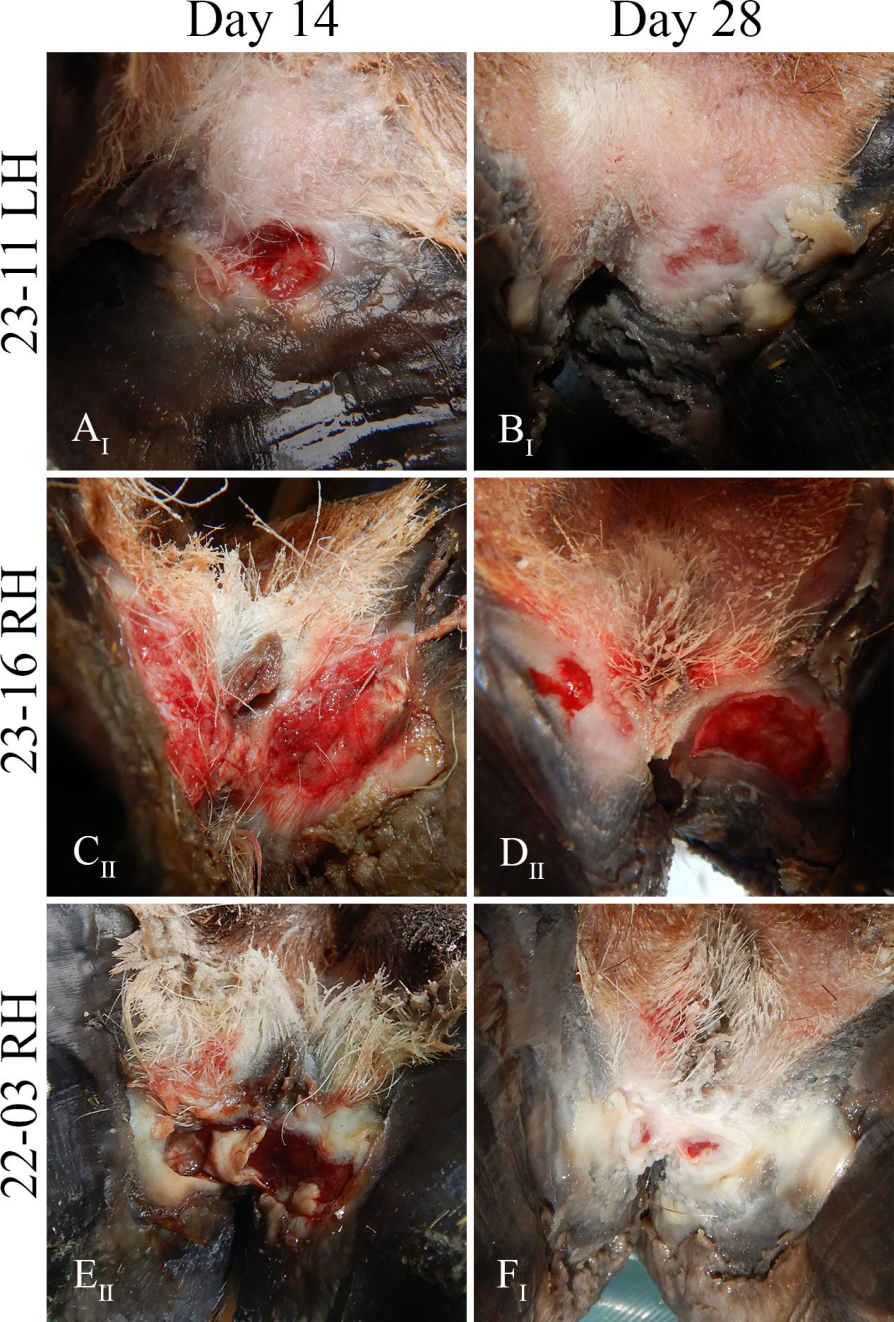
Representative photographs of experimentally induced lesions on the inoculated feet of three treatment elk (23-11, 23-16, and 22-03). The grade (I or II) assigned to each depicted lesion is denoted in the subscript of each photo. Grade I lesions observed on day 14 consisted of a focal discrete erosion on the interdigital skin (**A**) that remained relatively static through day 28 (**B**). Grade II lesions observed on day 14 were larger, extending beneath the hoof capsule and/or heel bulb (**C and E**). By day 28, previous grade II lesions were reduced in size (**D and F**) and reclassified as grade I lesions if the erosion no longer extended to the coronary band (**F**). RH, right hind foot. LH, left hind foot.

On day 14, interdigital erosions were observed on 8/10 inoculated feet from 4/5 treatment elk (Table 1). Lesions on three feet were categorized as grade II (Table 1) and consisted of approximately 4 cm × 1–1.5 cm erosions extending along the axial surfaces of both digits, disrupting the coronary band and adjacent hoof capsule and/or heel bulb (Fig. 5C and E). One foot with a grade II lesion (23-10 RH) also had a separate discrete, 3.0 × 1.0 cm ulcer resembling a grade I lesion on the haired interdigital skin at the site of prior abrasion. The remaining five affected feet had a focal, discrete, 0.3–1.5 cm diameter interdigital erosion at the site of prior abrasion, categorized as a grade I lesion (Table 1; Fig. 5A). Interdigital lesions were not detected on the hind feet of the control elk (Table 1).

On day 28, gross lesions were observed in all treatment elk, on 9/10 inoculated feet (Table 1). The grade I lesions remained unchanged between day 14 and day 28 (Fig. 5A and B). All previous grade II lesions were reduced in size and reclassified as grade I lesions when the erosion no longer extended to the coronary band (Table 1 and Fig. 5C through F). New grade II lesions appeared on two treatment elk feet (22-04 LH and 23-10 LH). No erosions were observed on the hind feet of the control elk on day 28, although focal hyperpigmentation of the interdigital skin was noted at the sites of previous abrasion.

**Fig 6:**
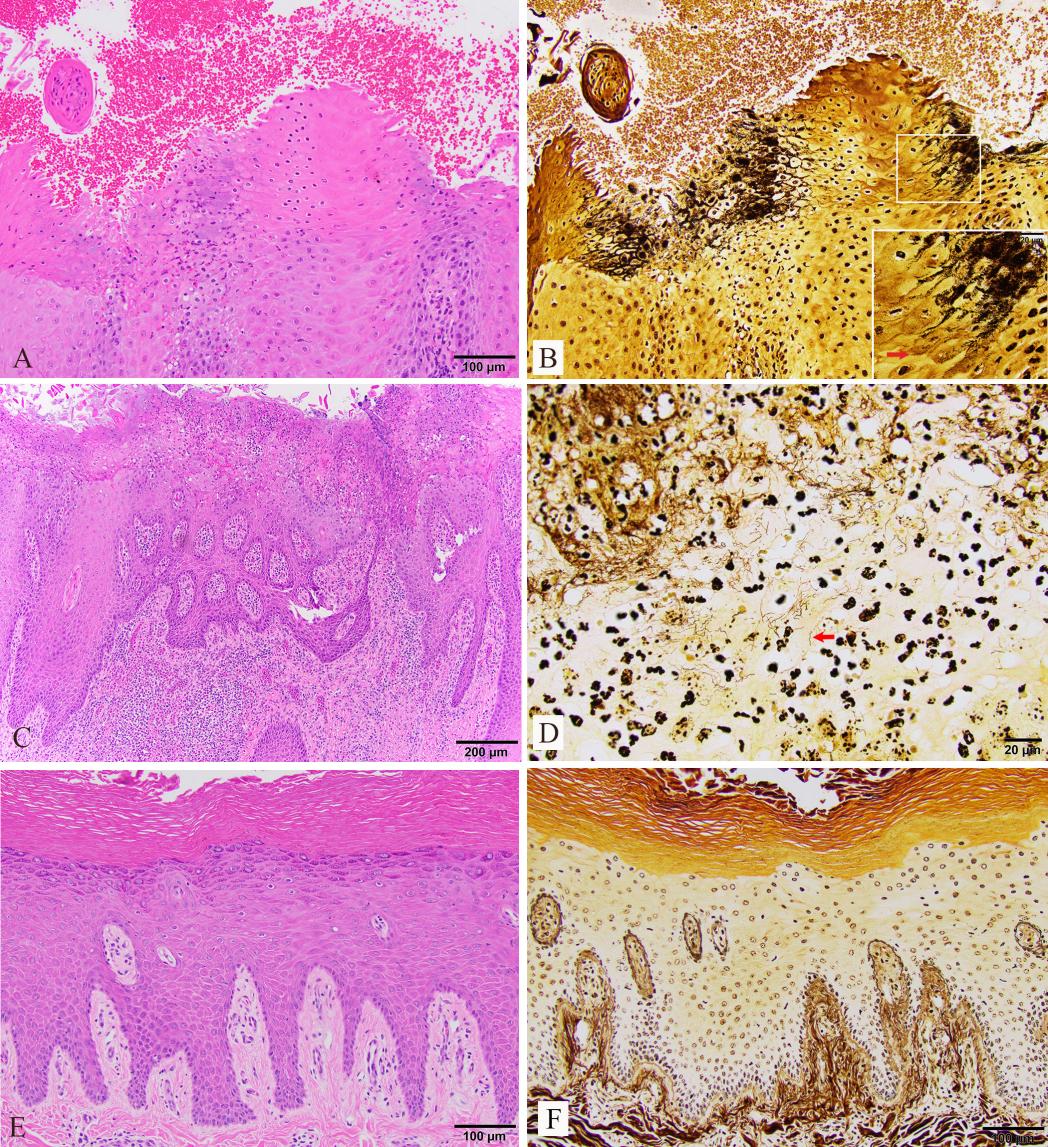
Representative photomicrographs of histologic lesions observed in samples of interdigital skin collected from the inoculated hind feet of treatment elk on day 28 (**A and B**) and days 41–44 (study conclusion; **C and D**). (**A and C**) At both time points, epidermal erosion and suppurative inflammation were apparent in H&E-stained skin sections. (**B and D**) Intralesional argyrophilic spirochetes (red arrows) were identified with a Warthin-Starry silver (WSS) stain. (B, inset) High-magnification view of spirochetes infiltrating between keratinocytes. Included for comparison are H&E-stained (**E**) and WSS-stained (**F**) sections of interdigital skin from the hind foot of a control elk without evidence of erosion, inflammation, or intralesional spirochetes.

A diagnosis of TAHD was confirmed histologically in biopsies collected on day 28 from 4/5 treatment elk and 6/9 hind feet with grade I or II gross lesions (Table 1). In biopsies from confirmed TAHD-positive feet, invasive argyrophilic spirochetes were identified within eroded and inflamed epidermal segments (Fig. 6A and B). Examination of a biopsy from the grade II lesion on elk 23-10 LH revealed a deep ulcer, but spirochetes could not be identified. A diagnosis of TAHD also could not be confirmed histologically for lesions on 23-16 LH and 22-04 LH, likely due to a suboptimal biopsy site. Foot biopsies from control elk were TAHD-negative (Table 1); although some contained mild chronic dermal inflammation, all had an intact epidermis and no apparent spirochetes.

At the conclusion of the study (days 41–44), histology confirmed TAHD in 8/10 inoculated feet from 5/5 treatment elk (Table 1; Fig. 6C and D). Three out of four feet with grade I or II lesions were confirmed TAHD-positive (Table 1). Although the six remaining treatment elk feet were assigned gross lesion grade 0, with only apparent healing antemortem biopsy sites visible (Table 1), five of these were histologically confirmed TAHD-positive based on the presence of suppurative inflammation and intralesional spirochetes (Table 1). Control elk remained histologically TAHD-negative (Table 1; Fig. 6E and F). For all elk, no gross or histologic lesions were observed on the uninoculated front feet at the study conclusion.

**Fig 7:**
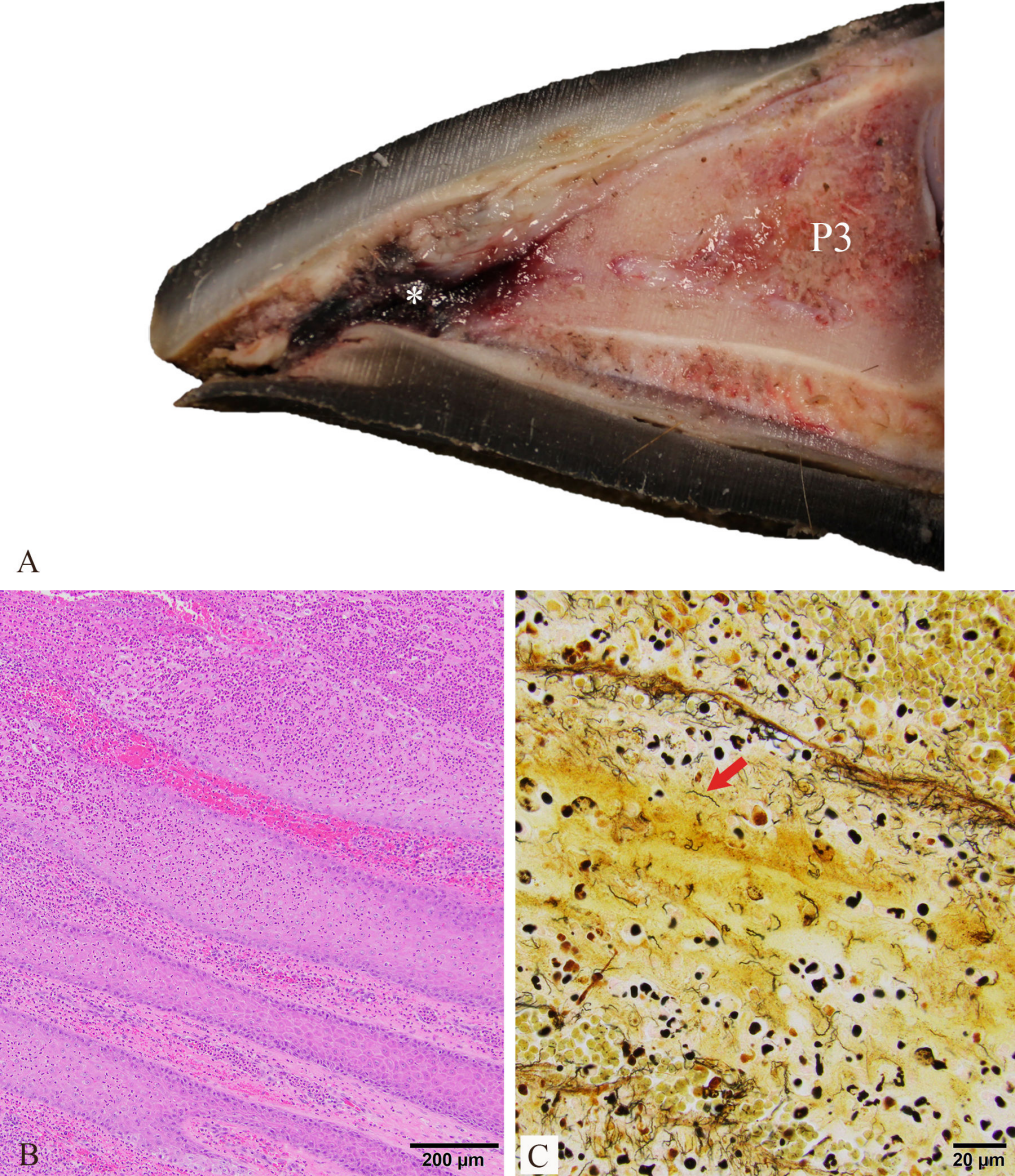
Toe tip lesion detected on the right hind foot of treatment elk 22-04 at the study conclusion. (**A**) Sagittal section of affected digit demonstrating hemorrhage and necrosis (asterisk) expanding the corium beneath the hoof wall and sole and abutting the third phalanx (P3). Image background removed in Microsoft Paint to improve lesion clarity. (**B**) There was extensive necrosuppurative inflammation and hemorrhage within the affected laminae on hematoxylin and eosin-stained histologic tissue sections. (**C**) Intralesional argyrophilic spirochetes (red arrow) were identified within the affected tissue using a Warthin-Starry silver stain.

In addition to the TAHD-positive interdigital skin lesion on elk 22-04 RH, this foot had focal separation of the sole along the white line at the distal aspect of the lateral digit. The exposed corium and epidermal laminae of the affected hoof were expanded and effaced by hemorrhage, necrosis, and suppurative inflammation, and numerous argyrophilic spirochetes were identified in the affected tissues with a Warthin-Starry silver stain, confirming a TAHD diagnosis (Fig. 7).

Postmortem examination revealed minimal tissue changes aside from the experimentally induced foot lesions. The popliteal lymph nodes from all treatment animals had follicular hyperplasia and sinusoids distended by leukocytes and erythrocytes, indicative of antigenic stimulation and localized hemorrhage and inflammation in the distal hind limbs.

### 16S amplicon sequencing of captive elk samples

#### Alpha diversity

**Fig 8:**
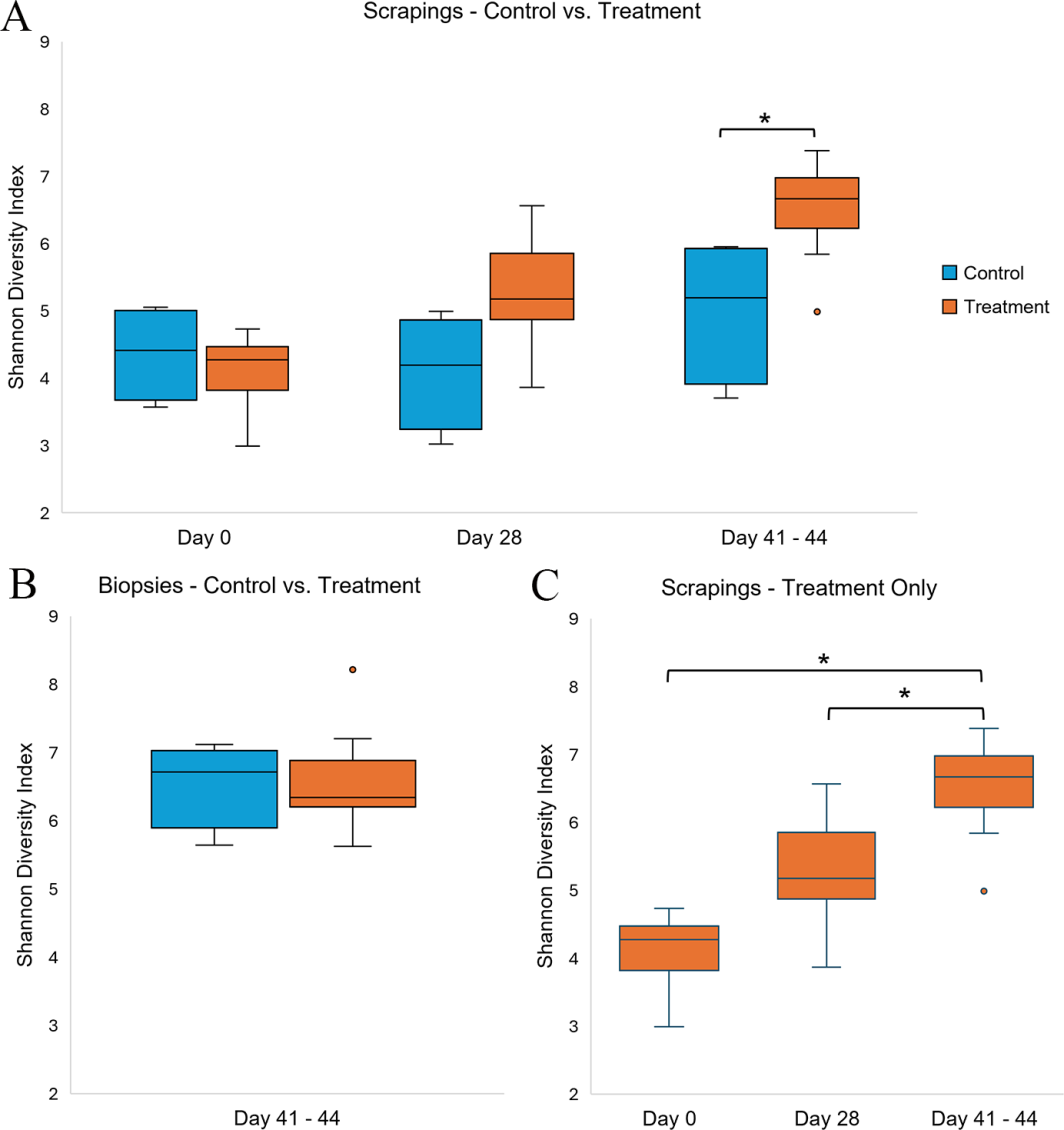
Boxplots comparing average SDI, a measure of alpha diversity, for samples collected from the hind feet of treatment and control elk at pre-inoculation (day 0) and post-inoculation (days 28 and 41–44) time points. Boxes extend from the 25th to 75th percentile, and horizontal lines denote the median. Outlier values >1.5× the interquartile range below the lower quartile or above the upper quartile are represented with blue or orange dots. (**A**) Comparison of SDI for control and treatment hind foot scrapings. Asterisk indicates time point with statistically significant differences between groups (*P* < 0.05) via Mann-Whitney *U* test. (**B**) There was no statistically significant difference in the SDI of hind foot biopsies collected from treatment and control elk at the study conclusion (days 41–44). (**C**) SDI comparison for treatment elk skin scrapings collected over the course of the study period. Treatment scrapings had a significantly higher SDI at days 41–44 compared to day 0 or 28 (*P* < 0.05; asterisks), as calculated with a Friedman test and *post hoc* pairwise comparisons using Wilcoxon signed-rank test with Bonferroni correction.

The difference in the average SDI between skin scrapings from treatment and control feet was not statistically significant on day 0 or 28 (Fig. 8A). Skin scrapings, but not biopsies, collected from treatment elk on days 41–44 had significantly higher SDI compared to the control group (*P* < 0.05; Fig. 8A and B). There was a significant effect of study day on the alpha diversity of skin scrapings collected from the hind feet of treatment elk (Friedman χ^2^ = 14.6, *P* < 0.001). The SDI increased post-inoculation, indicating an increase in within-sample bacterial diversity. Treatment foot scrapings had a significantly higher SDI at the study conclusion compared to day 0 or 28 (*P* < 0.05; Fig. 8C). Although scrapings from most inoculated treatment elk feet had a higher SDI on day 28 compared to day 0, the difference was not statistically significant (*P* = 0.057; Fig. 8C).

#### Bacterial community composition

**Fig 9:**
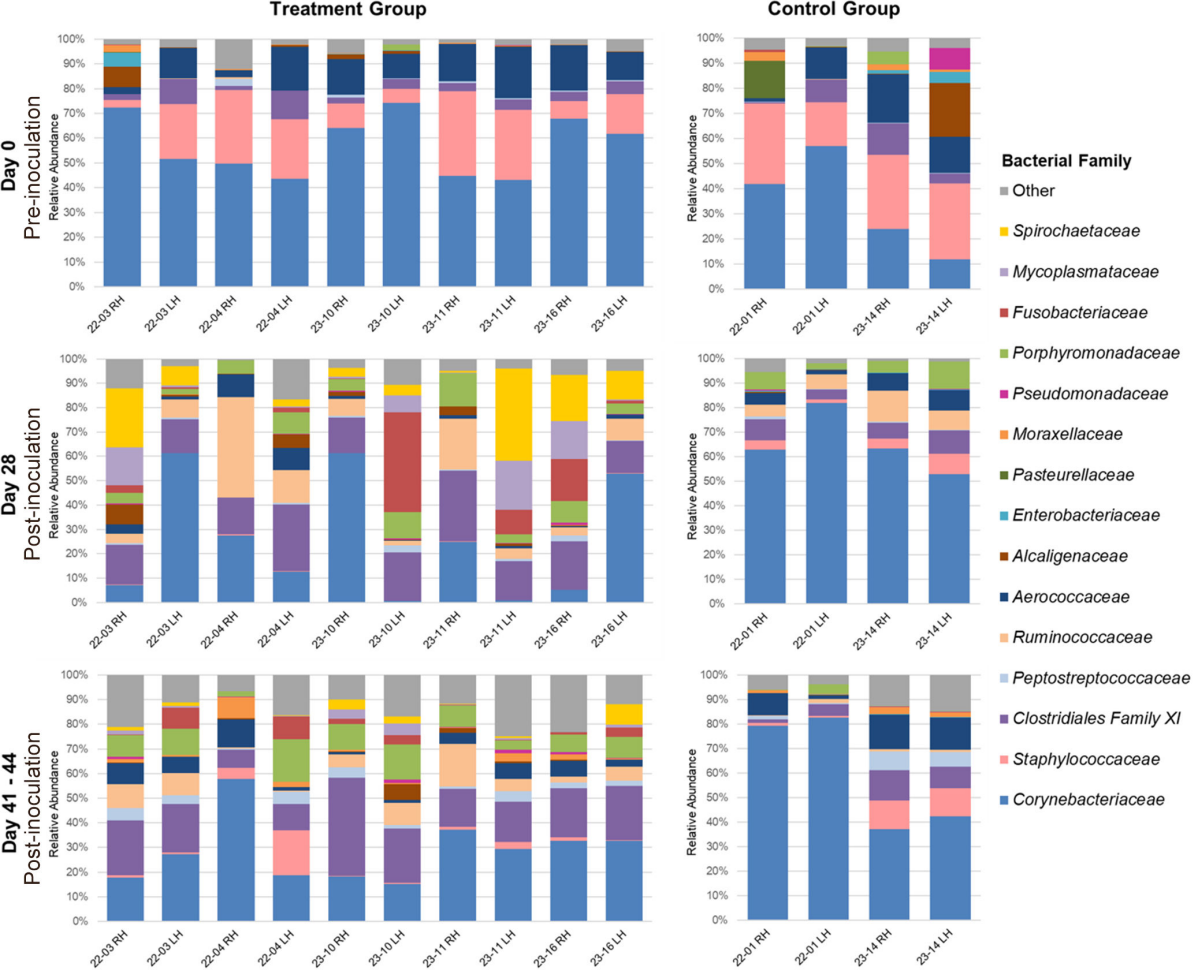
Family-level microbial composition of hind foot scrapings from treatment elk (left column) and control elk (right column) collected before (day 0) and after (day 28 and days 41–44) experimental inoculation, as determined by 16S rRNA gene amplicon sequencing. Bacterial families that were not detected at ≥5% relative abundance in at least one sample were grouped into the “other” category. RH, right hind foot. LH, left hind foot.

On day 0, the family-level bacterial community composition of foot scrapings from elk in both groups was similar, with a high RA of *Corynebacteriaceae* and *Staphylococcaceae* (combined RA = 42.1%–79.9%; Fig. 9). TAHD-associated taxa *Spirochaetaceae* and *Mycoplasmataceae* were not detected in any foot scrapings collected on day 0. *Fusobacteriaceae* was detected at low RA (<1.0%) in one day 0 foot scraping from a control elk.

**Fig 10:**
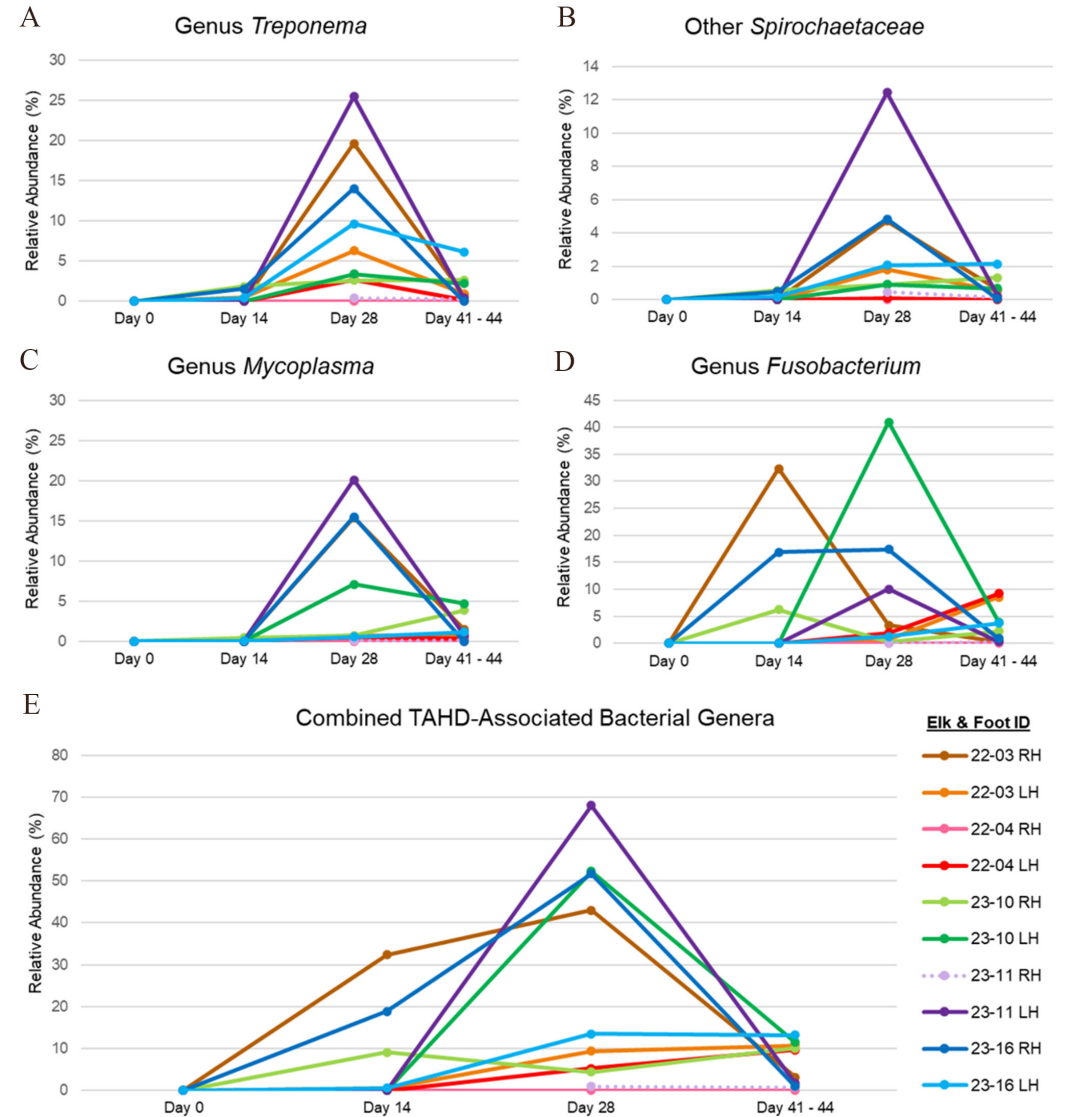
Percent RA of select elk hoof disease-associated bacterial genera in hind foot scrapings collected from treatment elk pre-inoculation (day 0) and post-inoculation (days 14, 28, and 41–44). These genera include *Treponema* (**A**), other unidentified *Spirochaetaceae* (**B**), *Mycoplasma* (**C**), and *Fusobacterium* (**D**). The bottom graph (**E**) depicts the combined RA of all four genera represented in graphs A–D. Taxonomic designations are based on 16S rRNA gene amplicon sequencing results using the Zymo Research reference database. The dashed line indicates that day 14 sequencing results were not available for 23-11 RH. RH, right hind foot. LH, left hind foot.

By day 14, *Treponema* was detected at a low RA (0.4%–1.8%) in the skin scrapings from 4/8 treatment elk feet with apparent grade I or II gross TAHD lesions (Table 1; Fig. 10A). *Fusobacterium* was also detected in 4/8 feet (RA <0.1%–32.4%; Fig. 10D), and *Mycoplasma* was detected in 2/8 feet (RA ≤0.5%; Fig. 10C) with lesions. These TAHD-associated bacterial genera were not detected in scrapings from control elk 23-14. Scrapings from three feet (control elk 22-01 RH and LH, and treatment elk 23-11 RH) did not yield 16S rRNA gene amplification.

**Fig 11:**
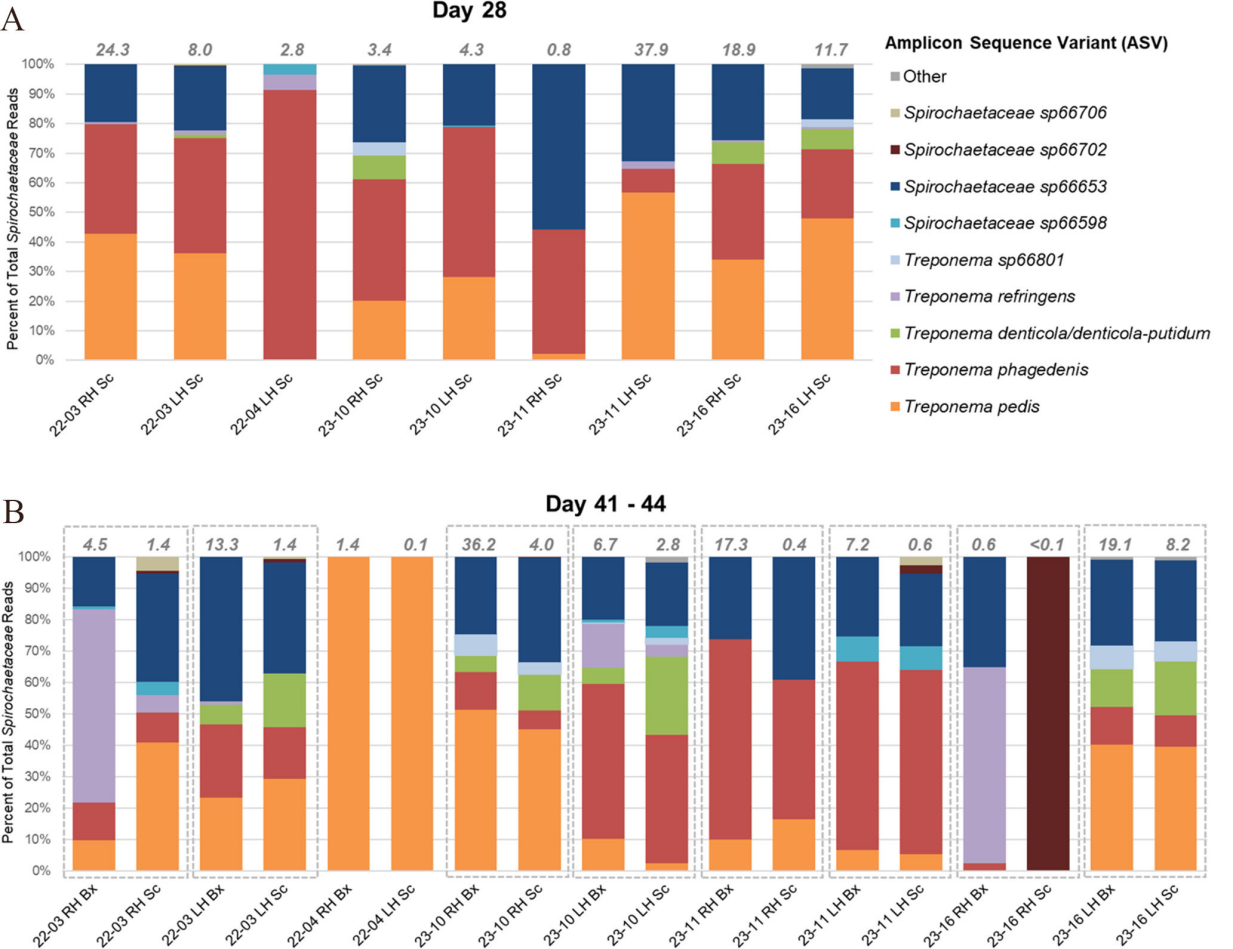
Relative proportion of ASVs in family *Spirochaetaceae* identified in post-inoculation scrapings (Sc) and biopsies (Bx) collected from the hind feet of treatment elk. Data are included for all samples in which *Spirochaetaceae* were detected by 16S rRNA gene amplicon sequencing on day 28 (**A**) and the conclusion of the study (days 41–44; **B**). Taxonomic designations are based on sequencing results using the Zymo Research reference database. ASVs that were not detected at ≥2% of total *Spirochaetaceae* reads in at least one sample were grouped into the “other” category. The percent relative abundance of family *Spirochaetaceae* in each sample is indicated by the italicized gray numbers at the top of each bar. Dashed gray boxes on graph B delineate paired postmortem scrapings and biopsies collected from the same foot. RH, right hind foot. LH, left hind foot.

By day 28, TAHD-associated bacterial taxa were detected in scrapings from the hind feet of all treatment elk. *Fusobacteriaceae* (genus *Fusobacterium;* RA <0.1%–41.0%), *Mycoplasmataceae* (genus *Mycoplasma;* RA = 0.1%–20.1%), *Treponema* spp. (RA = 0.3%–25.4%), and other *Spirochaetaceae* (RA = 0.1%–12.4%) were identified in scrapings from all nine treatment elk feet with graded gross lesions and collectively accounted for up to 68% of all bacterial reads in these samples (Table 1; Fig. 9 and 10). *T. pedis, T. phagedenis,* and *Spirochaetaceae* sp66653 were detected concurrently in 8/9 scrapings and collectively represented 87%–100% of all reads within family *Spirochaetaceae* (Fig. 11A). These TAHD-associated bacterial taxa were not detected in a scraping from the only inoculated treatment elk foot without a gross graded lesion on day 28 (22-04 RH).

At study conclusion (days 41–44), family *Spirochaetaceae* and genus *Treponema* were detected in biopsies and/or scrapings from both hind feet of all treatment elk, including feet with apparent resolution of prior gross lesions (Table 1). Compared to day 28, most endpoint scrapings had a lower RA of family *Spirochaetaceae* (Fig. 9 and 11) and specifically genus *Treponema* (Fig. 10). Most biopsies from the hind feet of treatment elk had a higher RA of *Spirochaetaceae* compared to matched scrapings, although the ASV-level composition of family *Spirochaetaceae* was similar for many of the paired samples (Fig. 11B). As on day 28, *T. pedis, T. phagedenis,* and *Spirochaetaceae* sp66653 were detected concurrently in most endpoint samples, collectively encompassing 37.6%–100% of all *Spirochaetaceae* reads (Fig. 11B). *Fusobacterium* and *Mycoplasma* were detected in all *Treponema*-positive endpoint biopsies and scrapings from the hind feet of treatment elk. Additionally, *T. pedis* and *Spirochaetaceae* sp66653 were detected along with *Mycoplasma*, *Fusobacterium,* and other anaerobes in samples of inflamed and necrotic tissue collected from the toe tip lesion on elk 22-04 RH (Table S5).

*Corynebacteriaceae* remained the bacterial family with the highest RA in hind foot samples from control elk following mock inoculation (Fig. 9). *Spirochaetaceae*, *Mycoplasmataceae,* and *Fusobacteriaceae* were not detected in hind foot scrapings collected from control elk on day 28 or 41–44 (Fig. 9).

Unidentified *Spirochaetaceae* not known to be associated with TAHD were detected at a low RA (≤0.1%) in biopsies from the front and hind feet of control elk collected at the study conclusion (Table S6). Similar *Spirochaetaceae* ASVs were also detected at low RA (≤0.3%) in a few samples from the uninoculated front feet of the treatment elk (Table S6), which served as within-elk negative control feet. *Fusobacteriaceae* was detected in a single front foot scraping from one treatment elk (RA <0.1%). *Mycoplasmataceae* was not detected in samples from the front feet of any elk.

### Spirochete cultivation from an experimentally induced lesion

At the conclusion of the study, anaerobic cultures were established from biopsies of one experimentally induced grade II lesion (23-10 LH) to recultivate spirochetes. At 2 days post-incubation, motile spirochetes were detected as a predominant bacterial population in examined wet mounts. Culture vials supplemented with FBS or RS each had marked turbidity at day 6 post-incubation, at which time wet mounts contained large numbers of mixed-morphology bacteria, including spirochetes.

Cultures supplemented with either FBS or RS had a high RA of *Spirochaetaceae* (49.6% or 70.2%, respectively) on day 2 post-incubation (Fig. S1A). The RA of *Spirochaetaceae* was markedly reduced (<8%) after 6 days of anaerobic incubation, coinciding with a substantially increased RA of *Porphyromonadaceae* (Fig. S1A). *T. phagedenis, T. medium/medium-vincentii,* and *Spirochaetaceae* sp66653 collectively constituted >99% of all reads within family *Spirochaetaceae* at both time points (Fig. S1B).

## DISCUSSION

Within the 7 week study period, all inoculated treatment elk (5/5) developed TAHD. Experimentally induced lesions were grossly and histologically consistent with mild to moderate TAHD lesions observed in free-ranging elk, including the presence of invasive argyrophilic spirochetes. Previously reported TAHD-associated bacterial genera *Treponema, Mycoplasma,* and *Fusobacterium* (1, 5) were concurrently detected in scrapings and/or biopsies from all inoculated treatment elk feet (10/10) by the conclusion of the study. In contrast, the mock-inoculated hind feet of control elk did not exhibit gross, histological, or microbiological evidence of TAHD.

Compared with the previously published TAHD transmission model in elk (4), the current approach required fewer inoculations (3 vs 8) applied over a shorter time course (14 vs 133 days) and prepared inoculum using smaller volumes of tissues acquired from fewer feet (7 vs 62) from fewer free-ranging elk (4 vs 16). These substantial reductions in required time and resources represent major methodological improvements over the previous model, especially given the logistical constraints associated with obtaining freshly harvested and expeditiously frozen elk feet for inoculum preparation.

We also observed shorter and less variable time to lesion onset following inoculation compared to the previous captive elk study (4). By day 14, interdigital erosions resembling grade I and II TAHD lesions were evident on 8/10 inoculated feet, progressing to 9/10 inoculated feet by day 28 (Table 1). Intralesional spirochetes were also detected in antemortem biopsies collected 4 weeks after initial inoculation. This timeline for lesion onset aligns with sheep exposed to TAHD lesion material (23) and is comparable to experimental induction of early digital dermatitis lesions in BDD models that used similar methods (24–27).

Grade I lesions frequently developed at abrasion sites, while more extensive grade II lesions occurred primarily in non-abraded regions of the interdigital skin (Fig. 5). Continued chafing of the moist, wrapped interdigital skin and pooling of inoculum along the plantar surface of the foot may have contributed to lesion development in these non-scraped regions. Consistent with previous TAHD and BDD infection models (23–27), abrasion and application of moist wraps appeared to promote rapid lesion development. Loss of foot wraps, both pre- and post-inoculation, was associated with delayed lesion onset in one treatment elk (22-04). Erosions were not observed on the hind feet of mock-inoculated control animals, supporting that while abrasion and application of moist wraps likely promoted rapid lesion development on treatment elk feet inoculated with infectious material, these factors alone were insufficient for lesion induction.

Compared to pre-inoculation scrapings and control elk foot scrapings, microbial diversity was increased within post-inoculation foot scrapings from treatment elk with TAHD lesions. This finding is consistent with previous observations of free-ranging elk with naturally occurring TAHD lesions (5). Similar to the previous elk TAHD transmission study (4), *T. pedis, T. phagedenis,* and *Spirochaetaceae* sp66653 (high sequence similarity to *T. medium;* Table S4) were the predominant spirochetes identified in antemortem, post-inoculation foot scrapings from treatment elk. Paired biopsy and skin scraping samples collected from treatment elk at the study conclusion showed similar detection rates of TAHD-associated bacterial taxa including *Treponema*, *Fusobacterium,* and *Mycoplasma* and appeared to capture similar spirochete profiles (Fig. 11B). Collectively, these results reinforce the potential utility of skin scrapings for future elk hoof microbiome studies, as reported previously (13), although a rigorous comparison of these sampling approaches is warranted.

Lesions in treatment elk did not progress in severity beyond grade II, and many gross lesions resolved by the conclusion of the study. Despite apparent resolution of gross lesions, 5/6 feet with assigned gross grade 0 were confirmed histologically as TAHD-positive at the study conclusion. Similar discordance between gross and histologic diagnoses has also been observed in natural cases of TAHD, suggesting the lack of sensitivity of macroscopic observations (1, 5). Lack of lesion persistence and progression may be related to the abbreviated study duration, limited number of inoculations, or other extrinsic or intrinsic factors influencing the host immune response. It is also possible that our treatment inoculum did not provide adequate or appropriately timed exposure to key bacteria necessary to perpetuate this suspect polybacterial disease. In particular, there was a lower RA of *Spirochaetaceae* within both the macerated tissue and culture-based inoculum administered on day 14 compared to earlier inoculations (Table S3), which could have limited subsequent lesion progression.

We used multiple techniques to assess the quality of treatment inoculum, each with different advantages and limitations. Through histology, we confirmed the presence of spirochetes within TAHD lesions used to prepare inoculum. 16S amplicon sequencing was used to characterize the bacterial composition of the inocula; however, these results were not available until after inoculum use and cannot assure bacterial viability. Darkfield or phase-contrast microscopy has been used previously to identify, enumerate, and assess the motility of spirochetes in cultures (6, 10, 32, 33), and in this study, similar evaluations facilitated the selection of optimal cultures for use in inoculum. However, the morphology of treponemes may be altered by culture conditions and duration (32, 34, 35), necessitating the use of additional diagnostics for definitive identification in real time. Development and validation of specific PCR probes for recently identified putative TAHD pathogens (5, 13) would allow for confirmation of pathogen presence in inoculum prior to use.

Incorporation of selective culture techniques has the potential to improve the quality of inoculum through enrichment for putative TAHD pathogens, including those with a low abundance in cultured tissues. This study represents a rare effort to cultivate treponemes from TAHD lesions, building on previous work by Clegg et al. (6) and demonstrating that viable treponemes may be cultivated from elk hoof tissues that are frozen for up to 8 months. The primary *Spirochaetaceae* ASVs detected in the enrichment cultures and culture-based inocula were *T. phagedenis* and *T. medium/medium-vincentii* (Fig. 4; Table S2)*,* which correspond with *Treponema* phylotypes previously cultivated from TAHD lesions (6). *T. pedis* was apparently poorly enriched (Table S2), despite representing >48% of *Spirochaetaceae* ASVs in biopsies of the cultured lesions (Table S1). *T. pedis* has not yet been successfully isolated from naturally occurring TAHD lesions and has fewer documented livestock-derived isolates compared to *T. phagedenis* and *T. medium* (6, 12). BDD treponemes differ in their ability to survive in various environmental and culture conditions, and interactions between different *Treponema* spp. may influence growth in mixed cultures (35, 36). Competition from other anaerobes, including *Fusobacteriaceae, Porphyromonadaceae*, and multiple families of *Clostridiales*, likely also impacted the growth of treponemes in the mixed anaerobic cultures used to prepare treatment inoculum. The inclusion of macerated tissues in the inoculum helped ensure exposure to diverse *Treponema* spp., including *T. pedis*, and other putative TAHD pathogens such as *Mycoplasma*, which were not enriched by our culture techniques. This underscores the importance of tissue-based inocula in experimental models of TAHD, similar to BDD models (24, 25).

We were able to recultivate multiple *Treponema* spp. present in the treatment inocula from one experimentally induced TAHD lesion, including *T. phagedenis*, *Spirochaetaceae* sp66653, and *T. medium/medium-vincentii* (Fig. S1B). Cultures of the experimentally induced lesion had a higher RA of *Spirochaetaceae* (Fig. S1A) compared to cultures of naturally occurring TAHD lesions (Table S2), which may be attributable to increased bacterial viability in fresh tissues and inhibition of overgrowth by other anaerobes due to improved antibiotic efficacy and a shortened incubation time.

Interestingly, one treatment elk (22-04) developed a necrotizing toe lesion on the lateral digit of the right hind foot (Fig. 7). The gross appearance resembled toe tip necrosis syndrome (aka toe abscesses or ulcers) in feedlot cattle, which typically occurs on the lateral digit of the hind feet and is attributed to excessive wear of the toe and damage of the white line by abrasive surfaces, which allow for bacterial entry into the affected claw (37, 38). A similar underlying pathogenesis related to loss of the protective foot covering and hoof abrasion due to fast pacing on exposed concrete flooring was suspected for the lesion observed in our study. We identified spirochetes in the affected hoof laminae using a Warthin-Starry silver stain (Fig. 7) and 16S amplicon sequencing (Table S5). BDD-associated treponemes have been identified in non-healing hoof lesions of dairy cattle, including toe ulcers/necrosis, and are speculated to represent opportunistic secondary infections of the traumatized hoof horn in BDD-endemic herds (37, 39, 40).

This study provides a model for rapid induction of grade I and II TAHD lesions in captive elk. Such mild TAHD lesions may be inconspicuous in free-ranging elk and challenging to detect in the field, especially if affected elk do not exhibit obvious hallmarks of disease such as hoof abnormalities or lameness (41, 42). Therefore, an experimental model that can reproduce early disease is particularly valuable, as it may help expand our understanding of the pathogens and environmental variables that initiate disease. Host factors such as age, sex, diet, and trace mineral deficiencies might also impact host immunity, disease susceptibility, and possibly recovery from minor lesions, and the experimental infection model can be used to control for and explore the effects of these variables. A better understanding of the factors contributing to the rapid resolution of experimentally induced lesions in this study can help not only improve the infection model but also provide additional insights into disease pathogenesis. Further modifications of the culture-based inoculum to select for specific putative bacterial pathogens may also provide additional information about disease etiology. Future experiments with larger numbers of captive elk can more fully elucidate the mechanisms by which TAHD is transmitted between conspecifics and determine the potential for transmission to other ruminants. Collectively, these additional insights could help inform management strategies for limiting disease spread and mitigating the negative impacts of TAHD in wild elk populations.

## Data Availability

V3-V4 16S rRNA gene amplicon sequences are available as fastq files within the NCBI Sequence Read Archive, BioProject ID PRJNA1357367. Supplementary data files include metadata and sequence read counts (RC) and relative abundance (RA) at multiple taxonomic levels for samples analyzed by 16S rRNA gene amplicon sequencing in this manuscript, including captive elk biopsies and scrapings (Data Set S1), biopsies of feet used to prepare inocula and subsamples of macerated tissue inoculum (Data Set S2), and cell pellets of enrichment cultures and pooled culture-based inocula (Data Set S3). The average Shannon diversity index (SDI) values used to compare the alpha diversity of captive elk samples are also available (Data Set S4).

## SUPPLEMENTAL MATERIAL

Data Set S1 spectrum.03822-25-s0001.xlsx Captive elk sample metadata and taxa detected using 16S amplicon sequencing.

Data Set S2 spectrum.03822-25-s0002.xlsx Inoculum metadata and taxa detected using 16S amplicon sequencing.

Data Set S3 spectrum.03822-25-s0003.xlsx Data from 16S amplicon sequencing of cultures.

Data Set S4 spectrum.03822-25-s0004.xlsx Shannon diversity index data for elk samples.

Supplemental Material spectrum.03822-25-s0005.docx Tables S1 to S6, Fig. S1, and captions for Data Sets S1 to S4.

ASM does not own the copyrights to Supplemental Material that may be linked to, or accessed through, an article. The authors have granted ASM a non-exclusive, world-wide license to publish the Supplemental Material files. Please contact the corresponding author directly for reuse.

## References

[1] Wild MA, Taylor KR, Shah DH, Garrison K, Mansfield K, Burco J, Winter SN, Drew ML, Han S, Bildfell R, Munk BA. 2022. Surveillance for an emergent hoof disease in elk (Cervus elaphus) in the US Pacific West supplemented by 16S rRNA gene amplicon sequencing. J Wildl Dis 58:487–499. doi:10.7589/JWD-D-21-00119 35417921

[2] Han S, Mansfield KG. 2014. Severe hoof disease in free-ranging Roosevelt elk (Cervus elaphus roosevelti) in southwestern Washington, USA. J Wildl Dis 50:259–270. doi:10.7589/2013-07-163 24484504

[3] Han S, Mansfield KG, Bradway DS, Besser TE, Read DH, Haldorson GJ, Alt DP, Wilson-Welder JH. 2019. Treponeme-associated hoof disease of free-ranging elk (Cervus elaphus) in southwestern Washington state, USA. Vet Pathol 56:118–132. doi:10.1177/0300985818798108 30244661

[4] Robinson ZB, Shah DH, Taylor KR, Wild MA. 2023. Transmission and lesion progression of treponeme-associated hoof disease in captive elk (Cervus canadensis). PLoS One 18:e0289764. doi:10.1371/journal.pone.0289764 37561744 PMC10414667

[5] Goldsmith EW, Taylor KR, Wild MA, Deb S, Sullivan T, Lofgren E, Garrison KR, Schroeder GM, Hilson C, Walrath NL, Burco JD, Lantz E, Winter SN, Shah DH. 2025. Bacterial community analysis of treponeme-associated hoof disease in free-ranging elk (Cervus canadensis): evidence for a polybacterial etiology with geographic consistency. Appl Environ Microbiol 91:e0088825. doi:10.1128/aem.00888-25 41042612 PMC12628766

[6] Clegg SR, Mansfield KG, Newbrook K, Sullivan LE, Blowey RW, Carter SD, Evans NJ. 2015. Isolation of digital dermatitis treponemes from hoof lesions in wild north American elk (Cervus elaphus) in Washington state, USA. J Clin Microbiol 53:88–94. doi:10.1128/JCM.02276-14 25355757 PMC4290963

[7] Wilson-Welder JH, Han S, Bayles DO, Alt DP, Kanipe C, Garrison K, Mansfield KG, Olsen SC. 2024. Correlation of lesion severity with bacterial changes in treponeme-associated hoof disease from free-roaming wild elk (Cervus canadensis). Anim Microbiome 6:20. doi:10.1186/s42523-024-00304-9 38650043 PMC11036743

[8] Evans NJ, Brown JM, Demirkan I, Murray RD, Vink WD, Blowey RW, Hart CA, Carter SD. 2008. Three unique groups of spirochetes isolated from digital dermatitis lesions in UK cattle. Vet Microbiol 130:141–150. doi:10.1016/j.vetmic.2007.12.019 18243592

[9] Evans NJ, Brown JM, Demirkan I, Murray RD, Birtles RJ, Hart CA, Carter SD. 2009. Treponema pedis sp. nov., a spirochaete isolated from bovine digital dermatitis lesions. Int J Syst Evol Microbiol 59:987–991. doi:10.1099/ijs.0.002287-0 19406779

[10] Sayers G, Marques PX, Evans NJ, O’Grady L, Doherty ML, Carter SD, Nally JE. 2009. Identification of spirochetes associated with contagious ovine digital dermatitis. J Clin Microbiol 47:1199–1201. doi:10.1128/JCM.01934-08 19204100 PMC2668357

[11] Sullivan LE, Clegg SR, Angell JW, Newbrook K, Blowey RW, Carter SD, Bell J, Duncan JS, Grove-White DH, Murray RD, Evans NJ. 2015. High-level association of bovine digital dermatitis Treponema spp. with contagious ovine digital dermatitis lesions and presence of Fusobacterium necrophorum and Dichelobacter nodosus. J Clin Microbiol 53:1628–1638. doi:10.1128/JCM.00180-15 25740778 PMC4400778

[12] Clegg SR, Carter SD, Birtles RJ, Brown JM, Hart CA, Evans NJ. 2016. Multilocus sequence typing of pathogenic treponemes isolated from cloven-hoofed animals and comparison to treponemes isolated from humans. Appl Environ Microbiol 82:4523–4536. doi:10.1128/AEM.00025-16 27208135 PMC4984274

[13] Deb S, Wild MA, LeClair T, Shah DH. 2024. Discovery of novel treponemes associated with pododermatitis in elk (Cervus canadensis). Appl Environ Microbiol 90:e0010524. doi:10.1128/aem.00105-24 38742897 PMC11218636

[14] Krull AC, Shearer JK, Gorden PJ, Cooper VL, Phillips GJ, Plummer PJ. 2014. Deep sequencing analysis reveals temporal microbiota changes associated with development of bovine digital dermatitis. Infect Immun 82:3359–3373. doi:10.1128/IAI.02077-14 24866801 PMC4136199

[15] Caddey B, Orsel K, Naushad S, Derakhshani H, De Buck J. 2021. Identification and quantification of bovine digital dermatitis-associated microbiota across lesion stages in feedlot beef cattle. mSystems 6:e0070821. doi:10.1128/mSystems.00708-21 34313462 PMC8409723

[16] Staton GJ, Angell JW, Grove-White D, Clegg SR, Carter SD, Evans NJ, Duncan JS. 2021. Contagious ovine digital dermatitis: a novel bacterial etiology and lesion pathogenesis. Front Vet Sci 8:722461. doi:10.3389/fvets.2021.722461 34631855 PMC8496452

[17] Dias AP, Orsel K, Gammariello CS, De Buck J. 2025. Sequential emergence and quantitative dynamics of key bacterial species preceding digital dermatitis lesion onset in dairy cattle. Vet Microbiol 302:110378. doi:10.1016/j.vetmic.2025.110378 39842366

[18] Nielsen MW, Strube ML, Isbrand A, Al-Medrasi WDHM, Boye M, Jensen TK, Klitgaard K. 2016. Potential bacterial core species associated with digital dermatitis in cattle herds identified by molecular profiling of interdigital skin samples. Vet Microbiol 186:139–149. doi:10.1016/j.vetmic.2016.03.003 27016768

[19] Caddey B, De Buck J. 2021. Meta-analysis of bovine digital dermatitis microbiota reveals distinct microbial community structures associated with lesions. Front Cell Infect Microbiol 11:685861. doi:10.3389/fcimb.2021.685861 34336713 PMC8322762

[20] Wong NST, Malmuthge N, Gellatly D, Nordi WM, Alexander TW, Ortega Polo R, Janzen E, Schwartzkopf-Genswein K, Jelinski M. 2024. Characterization of the hoof bacterial communities in feedlot cattle affected with digital dermatitis, foot rot or both using a surface swab technique. Anim Microbiome 6:2. doi:10.1186/s42523-023-00277-1 38254160 PMC10804539

[21] Winter SN, Fernandez MDP, Taylor KR, Wild MA. 2022. Associations between hair trace mineral concentrations and the occurrence of treponeme-associated hoof disease in elk (Cervus canadensis). BMC Vet Res 18:446. doi:10.1186/s12917-022-03547-3 36564777 PMC9783704

[22] Winter SN, Wild MA, Lantz EL, Hilson C, Watson KD, Yamauchi JM, Huyvaert KP. 2025. Liver mineral levels associated with hoof disease occurrence and severity in Roosevelt elk (Cervus canadensis) in California, USA. J Wildl Dis 61:357–369. doi:10.7589/JWD-D-24-00135 40026067

[23] Wilson-Welder JH, Mansfield K, Han S, Bayles DO, Alt DP, Olsen SC. 2022. Lesion material from Treponema-associated hoof disease of wild elk induces disease pathology in the sheep digital dermatitis model. Front Vet Sci 8:782149. doi:10.3389/fvets.2021.782149 35097043 PMC8790030

[24] Gomez A, Cook NB, Bernardoni ND, Rieman J, Dusick AF, Hartshorn R, Socha MT, Read DH, Döpfer D. 2012. An experimental infection model to induce digital dermatitis infection in cattle. J Dairy Sci 95:1821–1830. doi:10.3168/jds.2011-4754 22459830

[25] Krull AC, Cooper VL, Coatney JW, Shearer JK, Gorden PJ, Plummer PJ. 2016. A highly effective protocol for the rapid and consistent induction of digital dermatitis in Holstein calves. PLoS One 11:e0154481. doi:10.1371/journal.pone.0154481 27119564 PMC4847800

[26] Wilson-Welder JH, Nally JE, Alt DP, Palmer MV, Coatney J, Plummer P. 2018. Experimental transmission of bovine digital dermatitis to sheep: development of an infection model. Vet Pathol 55:245–257. doi:10.1177/0300985817736572 29145798

[27] Thomas AD, Pajor EA, Caddey B, Goldhawk C, Martins L, Orsel K. 2022. An experimental model to induce digital dermatitis in beef calves. BMC Vet Res 18:238. doi:10.1186/s12917-022-03345-x 35739561 PMC9219410

[28] Beninger C, Naqvi SA, Naushad S, Orsel K, Luby C, Derakhshani H, Khafipour E, De Buck J. 2018. Associations between digital dermatitis lesion grades in dairy cattle and the quantities of four Treponema species. Vet Res 49:111. doi:10.1186/s13567-018-0605-z 30373670 PMC6206660

[29] Demirkan I, Erdoğan M, Demirkan AÇ, Bozkurt F, Altındiş M, Navruz FZ, Köse Z. 2018. Isolation and identification of Treponema pedis and Treponema phagedenis-like organisms from bovine digital dermatitis lesions found in dairy cattle in Turkey. J Dairy Sci 101:10317–10326. doi:10.3168/jds.2017-14227 30219415

[30] Brodard I, Alsaaod M, Gurtner C, Jores J, Steiner A, Kuhnert P. 2021. A filter-assisted culture method for isolation of Treponema spp. from bovine digital dermatitis and their identification by MALDI-TOF MS. J Vet Diagn Invest 33:801–805. doi:10.1177/10406387211008511 33834899 PMC8229849

[31] Caporaso JG, Kuczynski J, Stombaugh J, Bittinger K, Bushman FD, Costello EK, Fierer N, Peña AG, Goodrich JK, Gordon JI, et al.. 2010. QIIME allows analysis of high-throughput community sequencing data. Nat Methods 7:335–336. doi:10.1038/nmeth.f.303 20383131 PMC3156573

[32] Schrank K, Choi BK, Grund S, Moter A, Heuner K, Nattermann H, Göbel UB. 1999. Treponema brennaborense sp. nov., a novel spirochaete isolated from a dairy cow suffering from digital dermatitis. Int J Syst Bacteriol 49 Pt 1:43–50. doi:10.1099/00207713-49-1-43 10028246

[33] Nally JE, Hornsby RL, Alt DP, Whitelegge JP. 2019. Phenotypic and proteomic characterization of treponemes associated with bovine digital dermatitis. Vet Microbiol 235:35–42. doi:10.1016/j.vetmic.2019.05.023 31282377 PMC6684396

[34] Döpfer D, Anklam K, Mikheil D, Ladell P. 2012. Growth curves and morphology of three Treponema subtypes isolated from digital dermatitis in cattle. Vet J 193:685–693. doi:10.1016/j.tvjl.2012.06.054 22901455

[35] Arrazuria R, Caddey B, Cobo ER, Barkema HW, De Buck J. 2021. Effects of different culture media on growth of Treponema spp. isolated from digital dermatitis. Anaerobe 69:102345. doi:10.1016/j.anaerobe.2021.102345 33596466

[36] Bell J, Crosby-Durrani HE, Blowey RW, Carter SD, Evans NJ. 2023. Survival of bovine digital dermatitis treponemes in conditions relevant to the host and farm environment. Anaerobe 82:102766. doi:10.1016/j.anaerobe.2023.102766 37479021

[37] Kofler J. 2017. Pathogenesis and treatment of toe lesions in cattle including “nonhealing” toe lesions. Vet Clin North Am Food Anim Pract 33:301–328. doi:10.1016/j.cvfa.2017.02.005 28579046

[38] Paetsch C, Fenton K, Perrett T, Janzen E, Clark T, Shearer J, Jelinski M. 2017. Prospective case-control study of toe tip necrosis syndrome (TTNS) in western Canadian feedlot cattle. Can Vet J 58:247–254. 28246411 PMC5302198

[39] Evans NJ, Blowey RW, Timofte D, Isherwood DR, Brown JM, Murray R, Paton RJ, Carter SD. 2011. Association between bovine digital dermatitis treponemes and a range of “non-healing” bovine hoof disorders. Vet Rec 168:214. doi:10.1136/vr.c5487 21493554

[40] Bay V, Griffiths B, Carter S, Evans NJ, Lenzi L, Bicalho RC, Oikonomou G. 2018. 16S rRNA amplicon sequencing reveals a polymicrobial nature of complicated claw horn disruption lesions and interdigital phlegmon in dairy cattle. Sci Rep 8:15529. doi:10.1038/s41598-018-33993-9 30341326 PMC6195575

[41] Wild MA, Sargeant GA, Garrison K, Conradson D. 2022. Association of antler asymmetry with hoof disease in elk. J Wildl Manag 86:e22245. doi:10.1002/jwmg.22245

[42] Winter SN, Fernández MDP, Clancey E, Garrison K, Mansfield K, Wild MA. 2023. Community science strategies reveal distributional patterns of treponeme‐associated hoof disease in Washington elk (Cervus canadensis). Transbound Emerg Dis 2023:6685108. doi:10.1155/2023/6685108 40303802 PMC12017226

