# Supplementary material for: An experimental infection model for rapid reproduction of treponeme-associated hoof disease in captive elk (Cervus canadensis)
Source: Microbiol Spectr. 2026 Apr 9;14(5):e03822-25. doi: 10.1128/spectrum.03822-25 (PMC13141939; doi:10.1128/spectrum.03822-25)
Supplement: Supplemental Material — Tables S1 to S6, Fig. S1, and captions for Data Sets S1 to S4. [file spectrum.03822-25-s0005.docx]

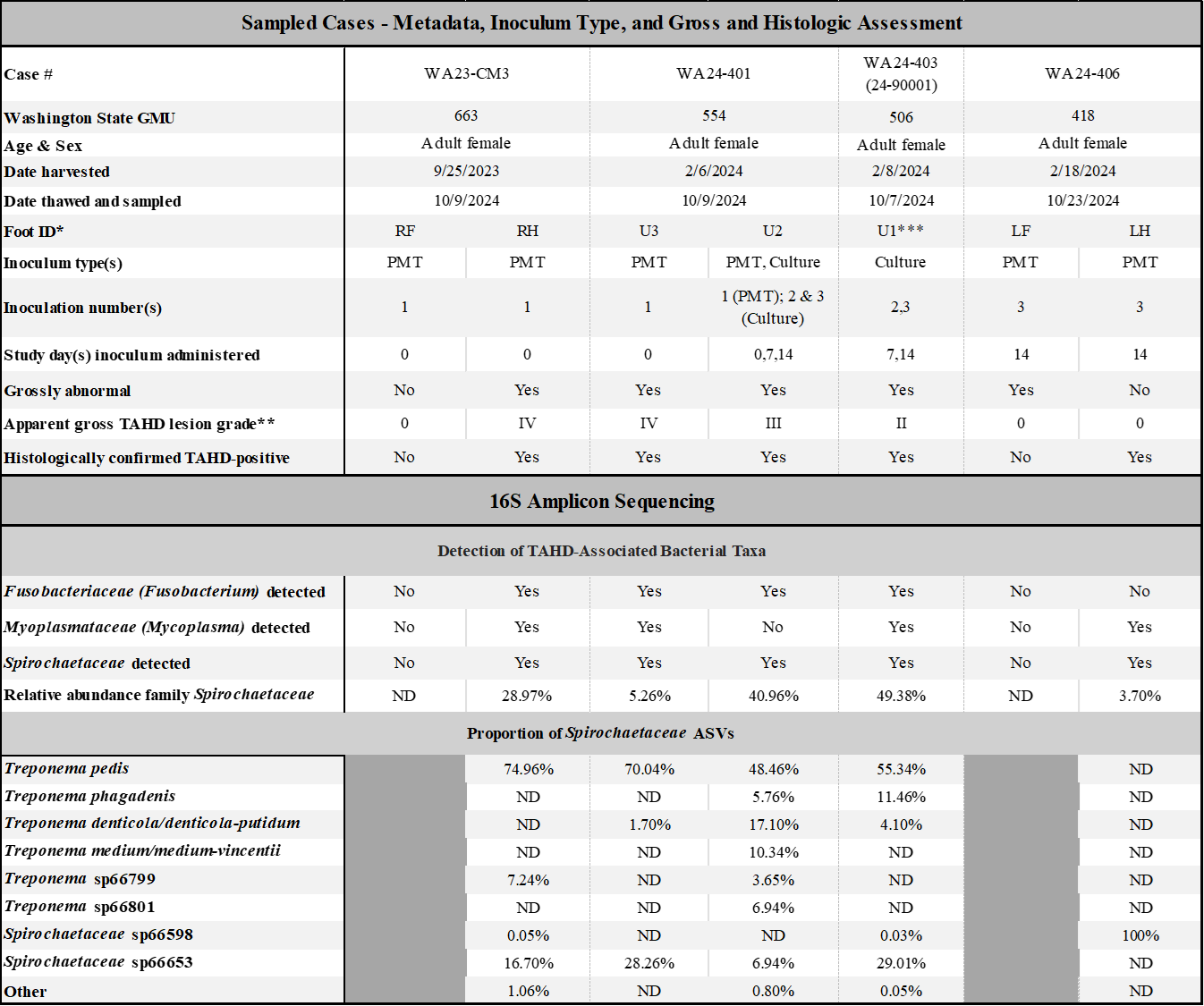
**Table S1:** Metadata, lesion detection, and limited 16S rRNA gene amplicon sequencing results for feet of free-ranging elk used to prepare all forms of treatment inoculum. Complete 16S sequencing results for these samples are available in Data Set S2.

*An arbitrary foot ID was assigned for internal use if not provided by the submitter.

**Gross lesion grade (0 – IV) assigned to each hind foot based on published TAHD grading scheme (1).

***16S sequencing was performed for two biopsies from this foot. *Spirochaetaceae* relative abundance and proportions of amplicon sequence variants (ASVs) only reported for sample with highest *Spirochaetaceae* relative abundance.

"Other" encompasses ASVs that comprised <2% of total *Spirochaetaceae* reads in all samples. Abbreviations: GMU = Game management unit. PMT = Pooled macerated tissue. TAHD = Treponeme-associated hoof disease. ND = Not detected.


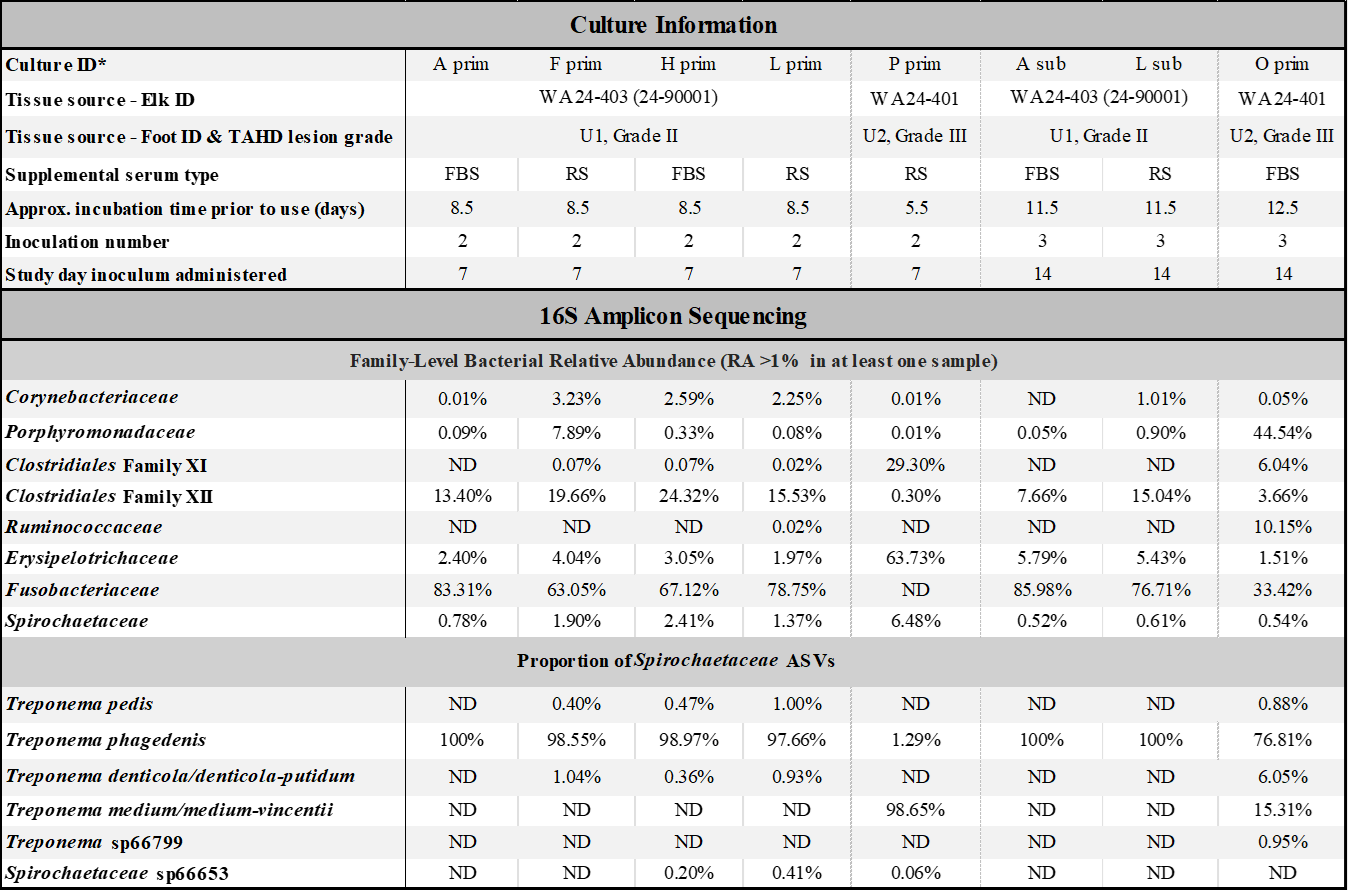


**Table S2:** Bacterial composition of individual anaerobic cultures used to prepare culture-based treatment inocula for application on days 7 and 14. Results are based on 16S rRNA gene amplicon sequencing of DNA extracted from cell pellets of each culture. Complete 16S sequencing abundance tables for these samples are available in Data Set S3.

*In culture ID, "prim" designates primary cultures, and "sub" designates subcultures. Abbreviations: TAHD = Treponeme-associated hoof disease. FBS = Fetal bovine serum. RS = Rabbit serum. ASV = Amplicon sequence variant. ND = Not detected.


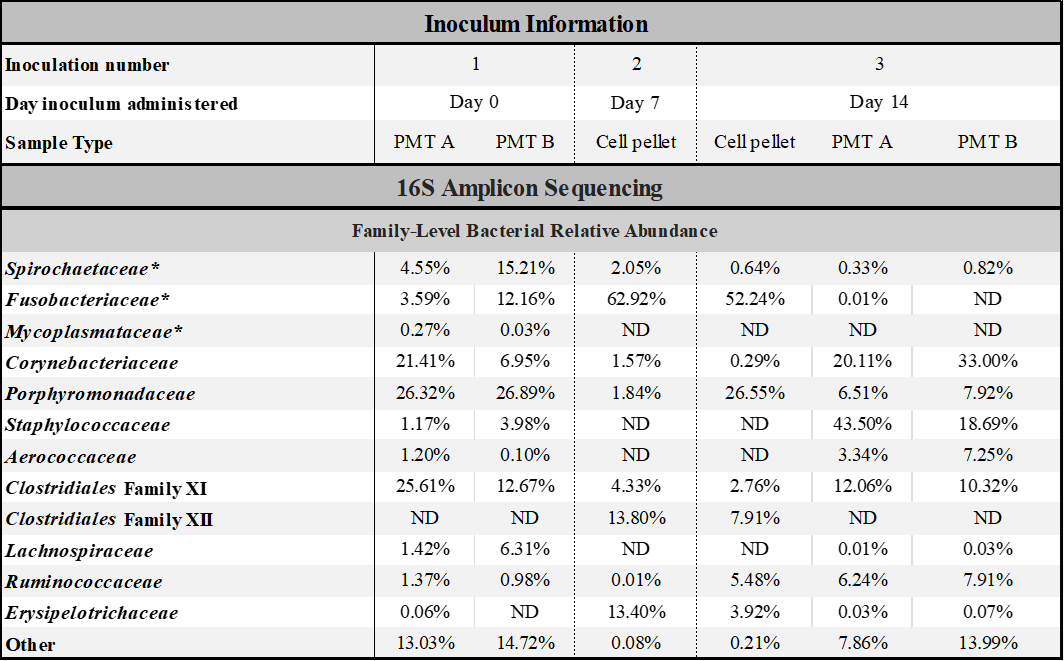


**Table S3:** Family-level bacterial composition of treatment inocula. DNA extraction and subsequent 16S rRNA gene amplicon sequencing was performed on two sample types: pooled macerated tissue (PMT - subsamples A and B) and cell pellets of enrichment culture-based inocula.

Asterisks indicate bacterial families associated with treponeme-associated hoof disease (TAHD). "Other" encompasses bacterial families that were not detected at ≥ 5% relative abundance in at least one sample. ND = Not detected. Complete 16S sequencing abundance tables for these samples are available in Data Set S2 and S3.


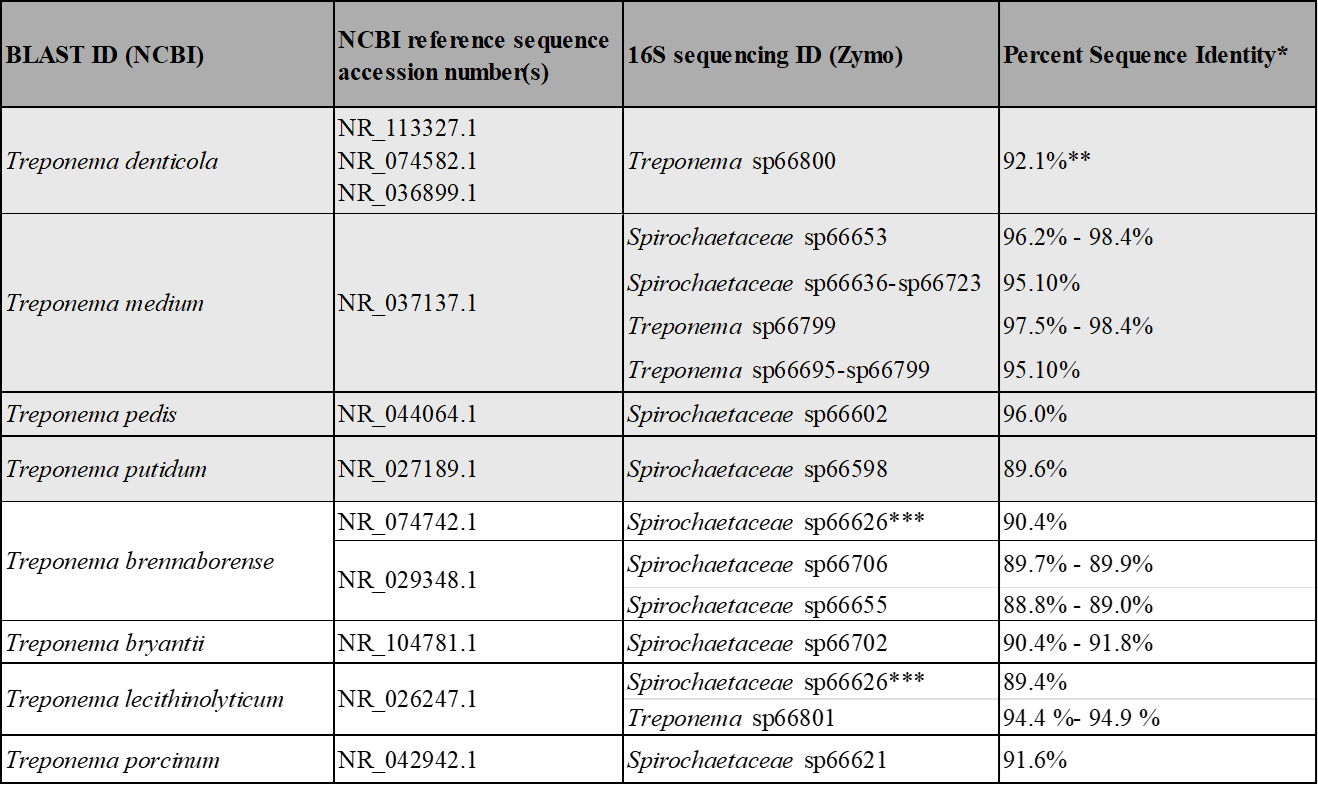


**Table S4:** Results of National Center for Biotechnology Information (NCBI) BLAST query (performed October 29-31, 2025) for 16S amplicon sequence variants (ASVs) assigned as unidentified *Treponema* spp. or *Spirochaetaceae* based on the Zymo Research reference database. Listed are accession numbers for the partial 16S rRNA reference sequences from the NCBI database with the highest percent identity for ASVs within each species-level designation. Specific *Treponema* phylotypes which have been associated with bovine digital dermatitis (BDD; 2) are indicated with gray shading.

*Range of percent identity is provided for all unique sequences assigned to each ASV, rounded to one decimal place.

**Equal percent identity for all reference sequences listed.

***Multiple sequences identified as *Spirochaetaceae* sp66626 had different matches on NCBI BLAST search.


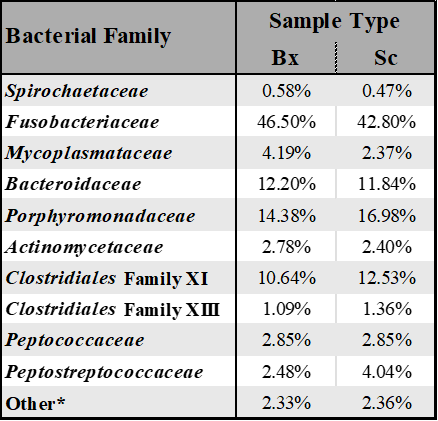


**Table S5:** Percent relative abundance of bacterial families identified within a biopsy (Bx) and scraping (Sc) of the toe tip lesion detected on the right hind foot of treatment elk 22-04 at the conclusion of the study, as determined by 16S rRNA gene amplicon sequencing.

*Encompasses other bacterial families that were detected at <1.0% RA in one or both sample types.

**Table S6:** Detection of *Spirochaetaceae* in scrapings (Sc) and biopsies (Bx) from uninoculated front feet and mock-inoculated hind feet of control elk at the study conclusion (day 41 – 44) via 16S rRNA gene amplicon sequencing.

Abbreviations: RA = Percent relative abundance based on total bacterial reads. ASV = Amplicon sequence variant. ND = Not detected.

**References for supplemental tables:**

1. Han S, Mansfield KG, Bradway DS, Besser TE, Read DH, Haldorson GJ, Alt DP, Wilson-Welder JH. 2019. Treponeme-associated hoof disease of free-ranging elk (*Cervus elaphus*) in southwestern Washington state, USA. Vet Pathol 56:118–132.
2. Evans NJ, Brown JM, Demirkan I, Murray RD, Vink WD, Blowey RW, Hart CA, Carter SD. 2008. Three unique groups of spirochetes isolated from digital dermatitis lesions in UK cattle. Vet Microbiol 130(1-2):141-50.


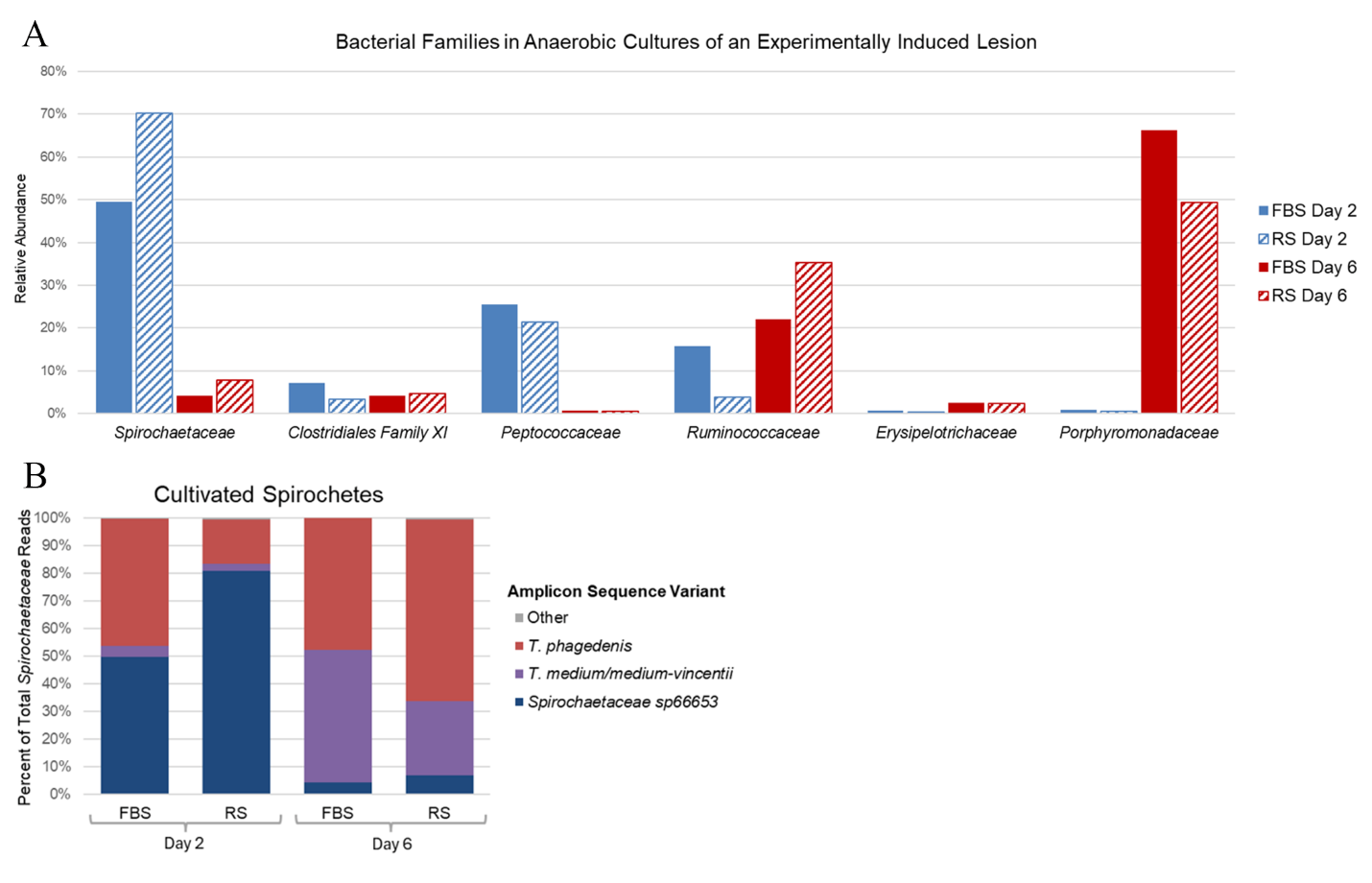


**Figure S1:** Bacterial composition of anaerobic cultures established from an experimentally induced, grade II treponeme-associated hoof disease (TAHD) lesion on the left hind foot of treatment elk 23-10. Cultures were initiated in oral treponeme enrichment broth supplemented with antibiotics and either fetal bovine serum (FBS) or rabbit serum (RS). Results are based on 16S rRNA gene amplicon sequencing of DNA extracted from cell pellets that were collected after 2 and 6 days of anaerobic incubation. (A) Relative abundance (RA) of bacterial families detected at >1% RA in cultures from either timepoint. Collectively, these families encompassed >99% of all bacterial reads in each sample. (B) Relative proportion of amplicon sequence variants (ASV) identified within family *Spirochaetaceae* in each cell pellet. "Other" represents ASVs detected at <1% of total *Spirochaetaceae* reads.

**Descriptions of supplemental data files (Excel sheets):**

**Data Set S1:** Metadata and 16S V3-V4 rRNA gene amplicon sequencing data for all captive elk samples analyzed, including read counts (RC) and relative abundance (RA) at multiple taxonomic levels.

**Data Set S2:** Metadata and 16S V3-V4 rRNA gene amplicon sequencing data for sampled feet from free-ranging elk used to prepare inocula and subsamples of the treatment and control pooled macerated tissue (PMT) inoculum.

**Data Set S3:**16S V3-V4 rRNA gene amplicon sequencing data for cell pellets of culture-based treatment inoculum prepared on days 7 and 14 and component anaerobic enrichment cultures used to prepare the inocula, and cell pellets of anaerobic cultures established from an experimentally induced, grade II treponeme-associated hoof disease (TAHD) lesion on the left hind foot of treatment elk 23-10.

**Data Set S4:** Average Shannon Diversity Index values for skin scrapings and biopsies collected from the hind feet of treatment and control elk on days 0 and 28 (scrapings) and day 41 – 44 (paired biopsies and scrapings).
